# Localized environmental heterogeneity drives the population differentiation of two endangered and endemic *Opisthopappus* Shih species

**DOI:** 10.1186/s12862-021-01790-0

**Published:** 2021-04-15

**Authors:** Hang Ye, Zhi Wang, Huimin Hou, Jiahui Wu, Yue Gao, Wei Han, Wenming Ru, Genlou Sun, Yiling Wang

**Affiliations:** 1 College of Life Science, Shanxi Normal University, Linfen, China; 2 Changzhi University, Changzhi, China; 3 Saint Mary’s University, Halifax, Canada

**Keywords:** *Opisthopappus*, Genetic differentiation, Environmental heterogeneity, Taihang Mountains

## Abstract

**Background:**

Climate heterogeneity not only indirectly shapes the genetic structures of plant populations, but also drives adaptive divergence by impacting demographic dynamics. The variable localized climates and topographic complexity of the Taihang Mountains make them a major natural boundary in Northern China that influences the divergence of organisms distributed across this region. *Opisthopappus* is an endemic genus of the Taihang Mountains that includes only two spatially partitioned species *Opisthopappus longilobus* and *Opisthopappus taihangensis*. For this study, the mechanisms behind the genetic variations in *Opisthopappus* populations were investigated.

**Results:**

Using SNP and InDel data coupled with geographic and climatic information, significant genetic differentiation was found to exist either between *Opisthopappus* populations or two species. All studied populations were divided into two genetic groups with the differentiation of haplotypes between the groups. At approximately 17.44 Ma of the early Miocene, *O. taihangensis* differentiated from *O. longilobus* under differing precipitation regimes due to the intensification of the Asian monsoon. Subsequently, intraspecific divergence might be induced by the dramatic climatic transformation from the mid- to late Miocene. During the Pleistocene period, the rapid uplift of the Taihang Mountains coupled with violent climatic oscillations would further promote the diversity of the two species. Following the development of the Taihang Mountains, its complex topography created geographical and ecological heterogeneity, which could lead to spatiotemporal isolation between the *Opisthopappus* populations. Thus the adaptive divergence might occur within these intraspecific populations in the localized heterogeneous environment of the Taihang Mountains.

**Conclusions:**

The localized environmental events through the integration of small-scale spatial effects impacted the demographic history and differentiation mechanism of *Opisthopappus* species in the Taihang Mountains. The results provide useful information for us to understand the ecology and evolution of organisms in the mountainous environment from population and species perspective.

**Supplementary Information:**

The online version contains supplementary material available at 10.1186/s12862-021-01790-0.

## Background

Understanding the processes that drive differentiation between populations and elucidating the mechanisms that underlie the origins and maintenance of genetic variations are major aims and fundamental tasks in evolutionary biology [1–5], which are also core issues in conservation biology [6, 7]. Myriad factors may impact the evolution and genetic differentiation of plant populations, where geological events and climate oscillations have been suggested as critical drivers [8–10]. In terms of geological events, mountain uplifts lead to complex topographies that can segregate large plant populations into multiple smaller sub-populations and enhance differentiation between species or populations through geographic isolation. Further, climatic oscillations can shift the ranges of species, resulting in novel environments with increased variability [11, 12]. To adapt to different environments, organisms evolve corresponding phenotypic variations and genetic differentiation [13, 14].

During this process, intensifying climate change has left an indelible imprint on the composition and divergence of populations or species [15, 16], which further greatly influenced the distribution patterns and shaped the genetic structures of populations [5, 17]. In general, the geographic processes of mountainous regions may influence the genetic makeup of plant populations over large spatial scales, whereas ecological processes from climate change may impact the genetic structures of plant populations at small spatial scales [5, 18–20].

The Taihang Mountains, with a north–south orientation (36–40 ºN, 112–115 ºE), are a prominent natural boundary in Northern China [21, 22], which have a geological developmental history of more than 2.5 billion years with a typical platform type crustal structure spanning the Mesoproterozoic to Paleozoic Eras. Their distinct geotectonic positioning has produced a unique geological and geomorphic landscape. The Southern Taihang Mountains have existed as a major boundary of neotectonic deformation, represented by the Yuntai Landform [21–23]. The Northern Taihang Mountains, represented by Zhangshiyan, Cnagyan, and Linlv Landforms, are higher than their southern counterparts with an average elevation of 1500 m [21, 24, 25].

Climatically, the southern region is home to a warm temperate semi-humid climate with a mean annual temperature of 12.7 °C and precipitation of 606.4 mm, while the northern region has a temperate continental monsoon climate with a mean annual temperature of ~ 10 °C and precipitation of 700 mm. The topographic complexity of the Taihang Mountains coupled with increasing climate variability have significantly impacted many organisms [21, 24]. Being an important germplasm resource, *Pyrus betulaefolia* exhibits abundant genetic diversity and variation, which might have been derived from the diversified environments of different populations located in the Taihang Mountains [26]. In contrast *Episyrphus balteatus* presented a non-obvious phylogeographical structure, which resulted from invalid geographical barriers of the Taihang and Yashan Mountains, where its population division was driven by the climatic changes following the uplift of the Taihang and Yashan Mountains [27]. The Taihang Mountains have been regarded as a distribution and diversity center for numerous genera [21, 24].

As an important perennial herbal germplasm resource of Asteraceae (*Opisthopappus*), which has been listed as a second-class protected plant in China [28], grows only on the steep slopes and cliffs of the Taihang Mountains that span Shanxi, Hebei, and Henan Provinces [28–30]. Being a diploid species (2n = 18) [31], comprised of *Opisthopappus taihangensis* and *Opisthopappus longilobus* [25, 31, 32], this genus is endemic in China and possesses significant ornamental and medicinal value [25, 32, 33]. Between *O. taihangensis* and *O. longilobus*, the morphological distinctions are primarily manifested in the leaves and bracts. For the former, there is appressed puberulent on both surfaces of the leaves, two pinnatisect stem leaves, and no bracteal leaves. For the latter, which has hairless leaves, there is one pinnatisect, except the basal stem leaves, and a pair of bracteal leaves beneath the involucres. Furthermore, several other categories of morphological differentiation have been observed, such as the leaf pinnatisect, sparsely pubescent or glabrous surfaces, stoma size and density, pollen colpus depth, and ostiole density [32, 34, 35].

Meanwhile, genetic variations emerged between these two species, whether in the forms of nuclear molecular markers or chloroplast gene sequences [36–40]. Remarkably, phylogenetic analyses based on chloroplast microsatellites (cpSSR) and sequence related amplified polymorphisms (SRAP) revealed that neither *O. longilobus* nor *O. taihangensis* formed a monophyletic clade [37, 38]. In particular, some populations of *O. longilobus* always integrated with *O. taihangensis* populations. Nevertheless, the interspecific hybridization of these two species have never been reported.

In previous researches, it was revealed that geographical distance had significant correlations with genetic differentiation among populations across *Opisthopappus* species [37, 38]. However, precisely how the climatic heterogeneity of the Taihang Mountains influenced genetic differentiation in populations of this genus had not been addressed as yet. Consequently, we hypothesized that the differentiation between these two species would be a hierarchically comprehensive process that might be initially impacted by climate shifts, subsequently by the geographical topography of China, and finally by the environmental heterogeneity of the Taihang Mountains.

For the present study (according to the hypothesis above), the roles and influences of environmental factors on species and population differentiation were investigated through the combination of geographic and climatic data, using single nucleotide polymorphisms (SNP) and insertion-deletion (InDel) markers of nuclear genes developed by Chai et al. [41] based on transcriptome data. The aims of this study were to: (i) analyze the genetic variations between species and between all studied populations; (ii) investigate the evolutionary processes and histories of *O. taihangensis* and *O. longilobus* species; (iii) estimate the effects of geographical and climatic variables; (iv) identify the possible key environmental factors that drive this differentiation. These results evaluated the demographic dynamics of *Opisthopappus* during the evolutionary process, explored the underlying mechanisms of inter-/intra-species differentiation, and provided some clues for the investigation of additional plant species in the Taihang Mountains.

## Results

### Genetic variation of *Opisthopappus* populations

**Fig. 1:**
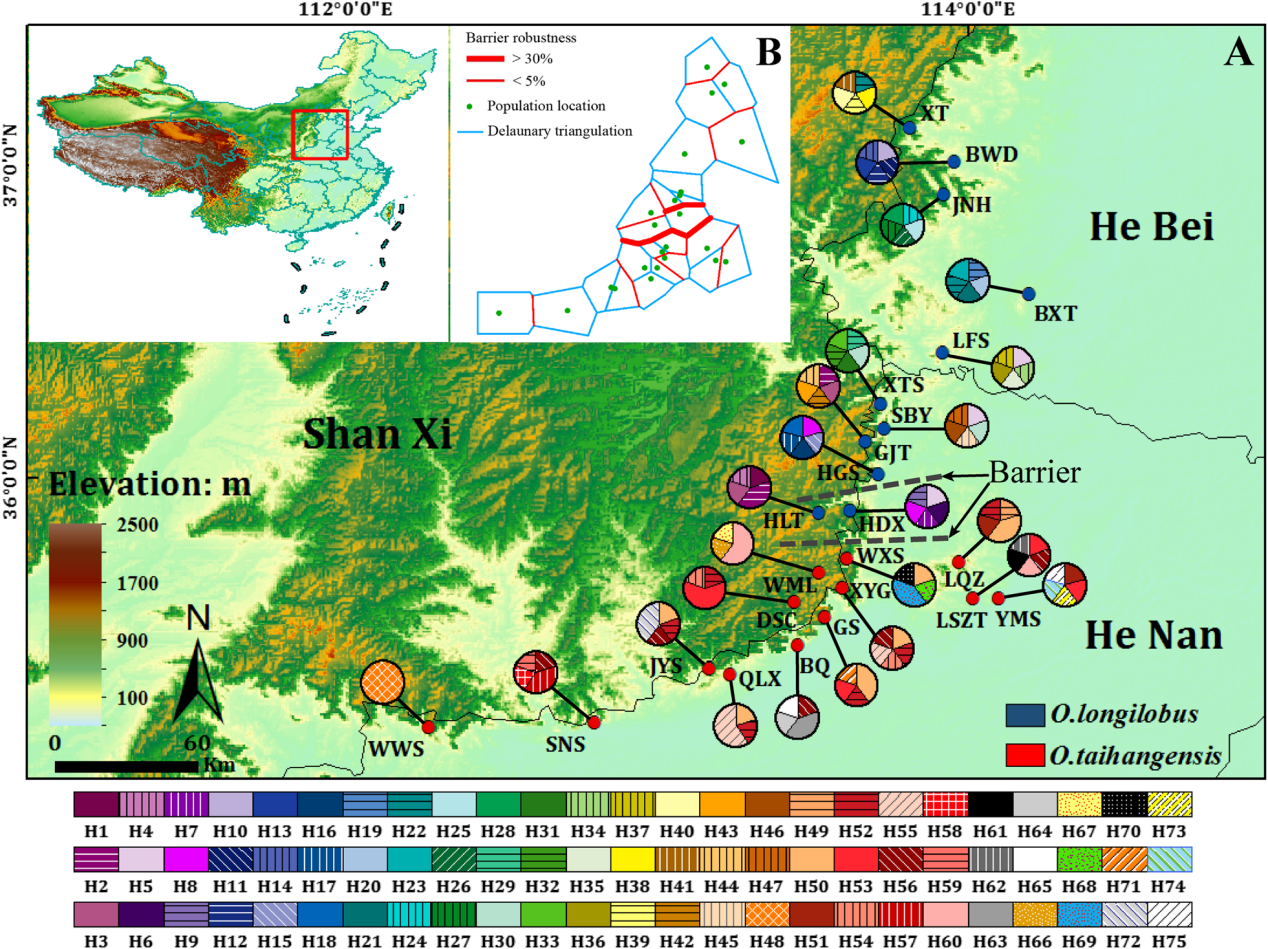
Sampling site with genetic boundary of *Opisthopappus* populations and distribution of haplotypes. **a** Distribution of 75 haplotypes detected within and among 24 populations of *O. longilobus* and *O. taihangensis*. Red circles refer to *O. taihangensis* populations and blue circles refer to *O. longilobus* populations, respectively. For population abbreviations, see Table 1 for details. **b** Results of the BARRIER analysis showed that the spatial separation of *Opisthopappus* populations. Delaunay triangulation and detected barrier (thick red line) separating *O. longilobus* and *O. taihangensis* in Taihang Mountains. Bootstrap values over 1000 replicates using Nei’s genetic distances

Among the SNP primers, eight pairs produced repeatable, clear, and stable bands. The total length of eight SNP combination segments was 1921 bp, which contained 1870 conservative sites and 51 polymorphic sites. Based on one hundred twenty sequences, seventy-five haplotypes were identified (Table 1). Therein, 47 haplotypes (H1–H47) were detected in *O. longilobus* and 28 haplotypes (H48–H75) in *O. taihangensis*. No shared haplotypes were detected between *O. longilobus* and *O. taihangensis*. For *O. longilobus*, the H5 haplotype was the most widely distributed, which was shared by three populations. Five haplotypes (H2, H3, H8, H22, and H30) were shared by two populations, whereas the other 41 haplotypes were distributed only among a single population (Table 1, Fig. 1). For *O. taihangensis*, the H50 and H52 haplotypes were the most widely distributed (both shared by six populations), followed by H56 (shared by five populations), H53 (shared by four populations), and H51, 54, 55, 60 (shared by two populations). The remaining 20 haplotypes were detected only in a single population.

**Table 1:** Sample location and genetic diversity estimation of *Opisthopappus* populations

Species	Population	Individual number (InDel/SNP)	Location	Longitude (E)	Latitude (N)	Altitude (m)	Number hapelotypes	Haplotype diversity (*H*d)	Nucleotide diversity (*π*)	*N*_a_	*N*_e_	*H*	*I*	*PPL*
*O. l*	HLT	14^*^/5^#^	Heilongtan, Shanxi	113.49°	36.01°	1054	H1(1)H2(2)H3(1)H4(1)	0.9	0.00083	1.2	1.1031	0.0631	0.0971	20.00%
	HDX	22^*^/5^#^	Hongdouxia,Shanxi	113.52°	35.91°	1070	H5(1)H6(1)H7(1)H8(1)H9(1)	1	0.00198	1.2923	1.145	0.0912	0.1413	29.23%
	BWD	9^*^/5^#^	Beiwudang,Hebei	114.08°	37.06°	1160	H10(1)H11(1)H12(1)H13(1)H14(1)	1	0.00208	1.2	1.146	0.0803	0.1167	20.00%
	HGS	7^*^/5^#^	Honggushan,Henan	113.72°	36.00°	741	H8(1)H15(1)H16(1)H17(1)H18(1)	1	0.00135	1.2308	1.1051	0.0671	0.1056	23.08%
	BXT	18^*^/5^#^	Beixiangtang,Hebei	114.22°	36.59°	520	H19(1)H20(1)H21(1)H22(1)H23(1)	1	0.00271	1.1538	1.052	0.0362	0.0603	15.38%
	JNH	12^*^/5^#^	Jingnianghu,Hebei	113.98°	36.99°	650	H24(1)H25(1)H26(1)H27(1)H28(1)	1	0.00344	1.3231	1.1861	0.1118	0.169	32.31%
	XTS	13^*^/5^#^	Xiantaishan,Henan	113.73°	36.18°	873	H29(1)H30(1)H31(1)H32(1)H33(1)	1	0.00229	1.2308	1.1345	0.0798	0.1204	23.08%
	LFS	13^*^/5^#^	Lufengshan,Hebei	113.76°	36.49°	1020	H5(1)H34(1)H35(1)H36(1)H37(1)	1	0.00271	1.2923	1.156	0.0962	0.1473	29.23%
	XT	14^*^/5^#^	Xingtaidaxiagu,Hebei	113.94°	37.20°	640	H22(1)H38(1)H39(1)H40(1)H41(1)	1	0.00146	1.2769	1.1354	0.0843	0.131	27.69%
	GJT	14^*^/5^#^	Gaojiatai,Henan	113.68°	36.11°	805	H2(1)H3(1)H42(1)H43(1)H44(1)	1	0.00292	1.3385	1.1701	0.1063	0.1643	33.85%
	SBY	14^*^/5^#^	Shibanyan,Henan	113.72°	36.15°	720	H5(1)H30(1)H45(1)H46(1)H47(1)	1	0.00328	1.2615	1.1663	0.0959	0.1425	26.15%
							47	0.99327	0.00308	1.7692	1.2532	0.1695	0.2777	76.92%
*O. t*	WWS	9^*^/5^#^	Wangwushan,Henan	112.27°	35.19°	3000	H48(5)	0	0	1	1	0	0	0.00%
	LQZ	8^*^/5^#^	Linqizhen,Henan	113.94°	35.72°	873	H49(1)H50(2)H51(1)H52(1)	0.9	0.00167	1.2308	1.1234	0.074	0.1134	23.08%
	DSC	12^*^/5^#^	Dashuangcun,Shanxi	113.44°	35.56°	868	H52(1)H53(3)H54(1)	0.7	0.00115	1.2154	1.116	0.0709	0.1084	21.54%
	XYG	8^*^/5^#^	Xiyagou,Shanxi	113.60°	35.64°	1268	H50(1)H52(1)H54(1)H55(1)H56(1)	1	0.00094	1.1692	1.1172	0.0662	0.0971	16.92%
	SNS	9^*^/5^#^	Shennongshan,Henan	112.82°	35.21°	1028	H56(1)H57(2)H58(1)H59(1)	0.9	0.00172	1.2462	1.1249	0.0776	0.1197	24.62%
	LSZT	7^*^/5^#^	Linshizuting,Henan	114.01°	35.62°	530	H53(1)H56(1)H60(1)H61(1)H62(1)	1	0.00146	1.2	1.1277	0.0729	0.1084	20.00%
	BQ	14^*^/5^#^	Baoquanshuiku,Henan	113.49°	35.47°	895	H56(1)H63(2)H64(1)H65(1)	0.9	0.00115	1.3231	1.1926	0.1116	0.1676	32.31%
	WML	14^*^/5^#^	Wangmangling,Shanxi	113.58°	35.69°	1421	H60(3)H66(1)H67(1)	0.7	0.00083	1.2154	1.1292	0.0765	0.1148	21.54%
	WXS	11^*^/5^#^	Wanxianshan,Henan	113.61°	35.73°	958	H50(1)H68(1)H69(2)H70(1)	0.9	0.00094	1.2769	1.1457	0.0879	0.1348	27.69%
	QLX	12^*^/5^#^	Qinglongxia,Henan	113.17°	35.40°	841	H50(1)H52(1)H55(3)	0.7	0.00042	1.3385	1.1617	0.0999	0.1561	33.85%
	GS	13^*^/5^#^	Guanshan,Henan	113.54°	35.56°	609	H50(2)H52(1)H53(1)H71(1)	0.9	0.00479	1.2	1.0967	0.0601	0.0937	20.00%
	JYS	13^*^/5^#^	Jingyingsi,Henan	113.20°	35.39°	1006	H50(1)H52(1)H56(1)H72(2)	0.9	0.00146	1.1692	1.0946	0.0557	0.0847	16.92%
	YMS	12^*^/5^#^	Yunmengshan,Henan	114.09°	35.61°	1005	H51(1)H53(1)H73(1)H74(1)H75(1)	1	0.00198	1.1077	1.0395	0.0271	0.0443	10.77%
							28	0.95399	0.00178	1.7385	1.246	0.1592	0.2585	73.85%
*Op*							75	0.98571	0.0073	1.9385	1.3985	0.246	0.3887	93.85%

*O. l*, *O. longilobus*; *O. t*, *O. taihangensis*; *Op., Opisthopappus*; *Na*, observed number of alleles; *Ne*, effective number of alleles; *H*, Nei’s genetic diversity; *I*, Shannon’s

information index; *PPB*, the percentage of polymorphic loci

The value of the genetic differentiation coefficient *N*_ST_ was 0.743 across species for the haplotypes with SNP data, which was significantly larger than the value of *G*_ST_ = 0.105 (*P* < 0.05). This indicated that there were significant phylogeographic structures in the *Opisthopappus* genus.

Both the SNP sequences and InDel data revealed a high genetic diversity in *Opisthopappus* (Table 1). For *O. longilobus*, the haplotype diversity (*H*d) was 0.99327 and the total nucleotide polymorphism (*π*) was 0.00308 according to the SNP data. As relates to the InDel data, the Nei’s gene diversity index (*H*), polymorphic loci ratio (*PPL*), and Shannon’s polymorphism information index (*I*) were 0.1695, 76.92%, and 0.2777, respectively. For *O. taihangensis*, the genetic indices, *H*d*, π*, *H*, *PPL* and *I*, were 0.95399, 0.00178, 0.1592, 73.85% and 0.2585 respectively (Table 1).

Significant genetic variations occurred either between *Opisthopappus* populations or two species (Table 2). Based on the SNP sequences, 80% of the mutations was found between *O. longilobu*s and *O. taihangensis* (*F*_CT_ = 0.8003, P < 0.01), 15% of the molecular variations within the populations (*F*_ST_ = 0.8460, P < 0.01), and only 5% of the molecular variations between populations within species (*F*_SC_ = 0.2287, P < 0.01). For the InDel data, the genetic variation distribution trend was similar to the SNP sequences. The results verified that molecular variations existed primarily between the two *Opisthopappus* species (Table 2).

**Table 2:** Analysis of molecular variance (AMOVA) based on pairwise differences for *Opisthopappus*

Source	df	SS	MS	Est. Var	%	Fixation Indices
SNP						
Among species	1	548.9707	548.9707	9.3417	80%	*F*_CT_ = 0.8003
Among populations within species	22	96.6499	4.3932	0.5331	5%	*F*_SC_ = 0.2287
Within populations	93	167.2000	1.7978	1.7978	15%	*F*_ST_ = 0.8460
Total	116	812.8205		11.6727	100%	(P < 0.001)
InDel						
Among species	1	321.7912	321.7912	5.2026	44%	*F*_CT_ = 0.4364
Among populations within species	22	402.0003	18.2727	2.9867	25%	*F*_SC_ = 0.4444
Within populations	93	347.2000	3.7333	3.7333	31%	*F*_ST_ = 0.6869
Total	116	1070.9915		11.9226	100%	(P < 0.001)

*F*_CT_, genetic differentiation among groups

*F*_SC_, genetic differentiation among populations within groups

*F*_ST_, genetic differentiation among populations

A maximum likelihood (ML) phylogenetic tree was constructed based on eight SNP combination fragments (Additional file 1: Fig. S1A), which revealed that all individuals were clearly divided into two groups corresponding to two species. Further, UPGMA cluster analysis (Additional file 1: Fig. S1B) performed based on Nei's genetic distance revealed that twenty-four populations were separated into two (*O. longilobus* and *O. taihangensis*) clusters.

**Fig. 2:**
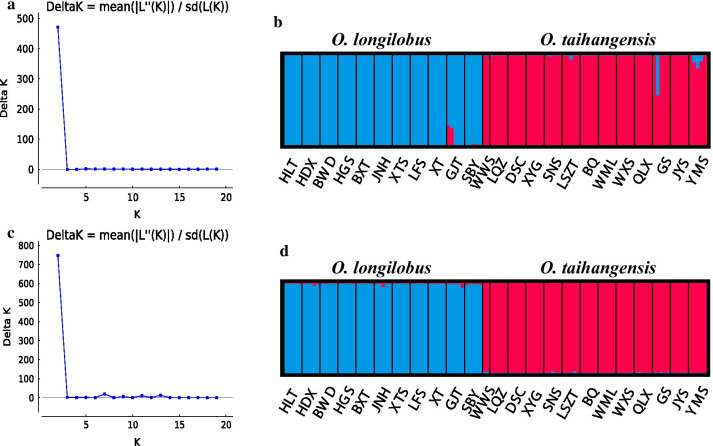
Results of the Bayesian clustering analysis conducted using STRUCTURE. The *∆K* plot conducted by Structure Harvester showed that *K* = 2 obtained the highest *∆K* value. **a** *∆K* plot of SNP. **b** Estimated genetic structure for *K* = 2 based on SNP. **c** *∆K* plot of InDel. **d** Estimated genetic structure for *K* = 2 based on InDel

For the structural analysis, when △*K* (mean (|L’(K)|/sd(L(K))) attained a maximum value, *K* = 2 was taken on both SNP and InDel data. The most significant possibilities were gathered into two groups (Fig. 2). When *K* ranged from 3 to 6, the genetic structural pattern was similar to that when *K* = 2, only more mixed individuals presented within *O. longilobus* or *O. taihangensis* populations (Additional file 2: Fig. S2).

**Fig. 3:**
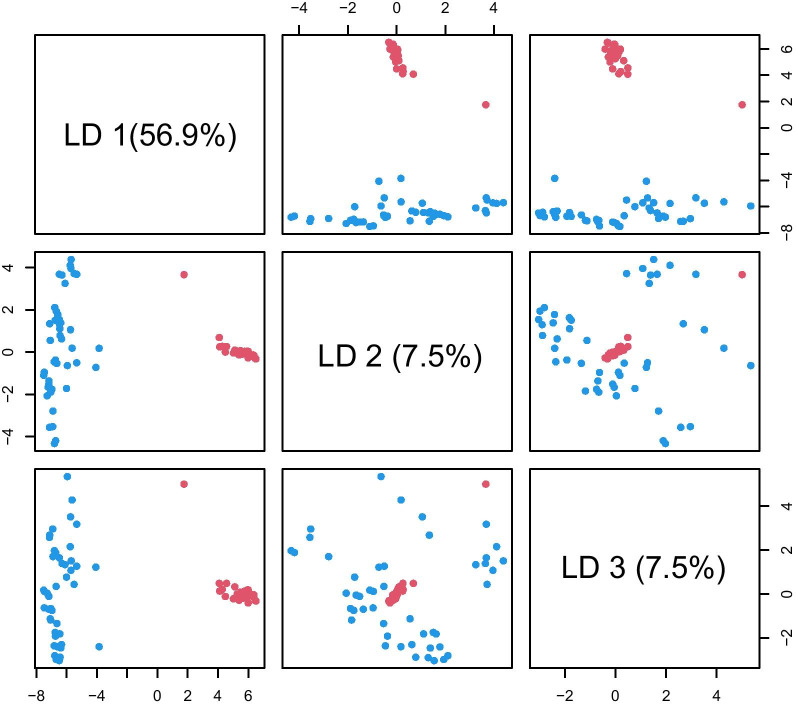
Genetic structure revealed by the discriminant analysis of principal components (DAPC). The DAPC for separating the species *O. longilobus* (blue) and *O. taihangensis* (red)

By the DAPC analysis, the conserved first ten principal components (PCs) represented a 91.1% variation of the total genetic components. The first three linear discriminant functions (LDs) explained 56.9%, 7.5%, and 7.5% of the eigenvalues of the remaining PCs. All *O. longilobus* and *O. taihangensis* populations could be thoroughly separated based on LD1 *&* LD2. From the plotting of LD2 *&* LD3, the two species were seemingly not well separated, which was attributed to the limited eigenvalues of variation proportion contained by LD2 and LD3 (Fig. 3). The results were confirmed by the K-W test (Additional file 3: Fig. S3).

### Historical dynamics of *Opisthopappus* populations

**Fig. 4:**
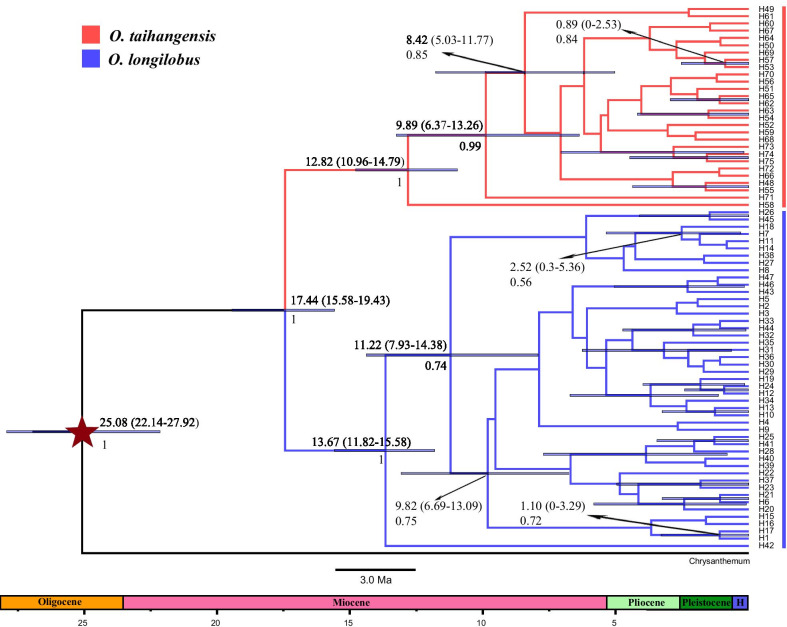
BEAST-derived chronogram of *Opisthopappus* based on haplotypes. The red branches represent haplotypes of *O. taihangensis* and the blue branches represent haplotypes of *O. longilobus*. Blue bars at nodes represented the 95% highest probability density (HPD) for the age of that node. The estimated divergence time (indicated by a five-pointed star) referred to Time Tree (http://www.timetree.org/) of the most recent common ancestor (tMRCA) of *Opisthopappus* and the outgroup was employed for calibration. Divergence times with 95% HPD and posterior probabilities (> 0.5) were labeled above and under nodes, respectively

A Bayesian inference tree was developed for seventy-five haplotypes (Fig. 4), which were segregated within two distinct branches. One branch contained the H1-H47 haplotypes from *O. longilobus*, whereas the other contained the H48-H75 haplotypes from *O. taihangensis*. Further, the haplotype network (Additional file 4: Fig. S4) presented a similar haplotype distribution pattern within the haplotype phylogenetic tree (Fig. 4).

At the Miocene-Oligocene boundary (~ 25.08 Ma, 95% HPD: 22.14–27.92 Ma), the *Opisthopappus* genus diverged from the outgroup (Fig. 4). In early Miocene, approximately 17.44 Ma (95% HPD: 15.58–19.43 Ma), *Opisthopappus* began to differentiate into two major lineages (*O. longilobus* and *O. taihangensis*). Within *O. taihangensis*, the differentiation time was at 12.82 Ma (95% HPD: 10.96–14.79 Ma) between haplotypes, while the haplotypes of *O. longilobus* were differentiated at ~ 13.67 Ma (95% HPD: 11.82–15.58 Ma). From the later Miocene to Pliocene, the intraspecies divergence continuously occurred, where the approximate more recent differentiation time of the intraspecific haplotypes for both species was during the Quaternary Era (e.g. H1 and H17, H53 and H57).

The neutral test and mismatch distribution analysis (MDA) (Additional file 5: Table S1) suggested that both *Opisthopappus* genus and two species had experienced a recent expansion based on significantly negative Fu's *Fs* (− 25.2963, − 18.5566, and − 24.1000 for *O. longilobus*, *O. taihangensis*, and *Opisthopappus*, respectively, P < 0.05) values and a non-significant sum of square deviation (SSD) and raggedness index (Rag) values (P > 0.05).

According to ABC analysis, there was a significant difference between the observed and simulated data based on the posterior distributions of all scenarios. Scenario 3, namely, *O. longilobus* was ancestral, and *O. taihangensis* differentiated from *O. longilobus*, was considered to be the most unambiguously supported evolutionary model with the highest posterior probability under direct estimate (0.3900, 95% CI 0.0000–0.8175) and logistic regression tests (0.7187, 95% CI 0.7093–0.7282). Furthermore, a 95% CI of the PP for this scenario did not overlap with other scenarios under logistic estimation.

**Fig. 5:**
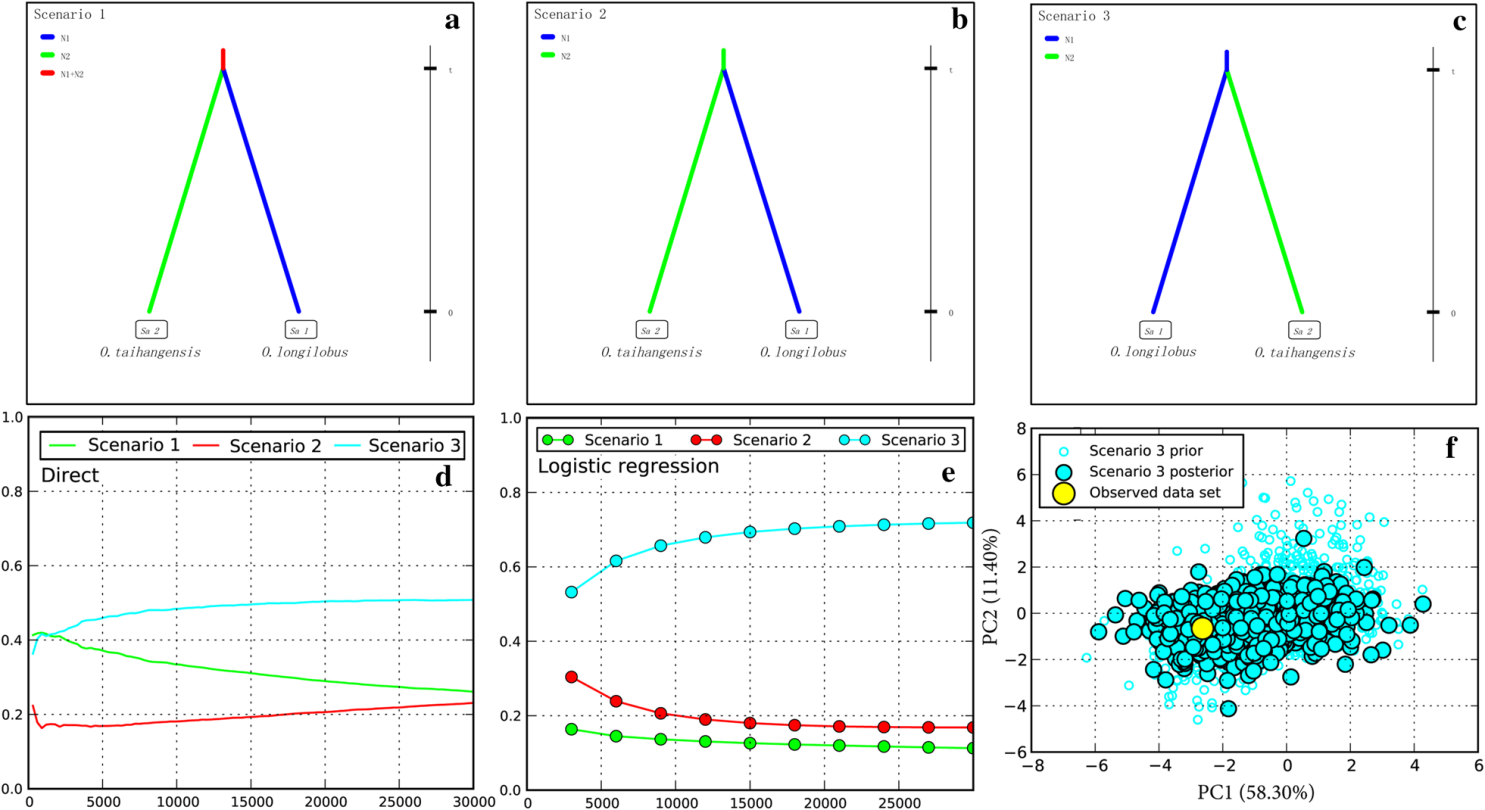
Summary results of the Approximate Bayesian Computation analysis of *Opisthopappus* assessed using DIYABC software analyses based on *K* = 2. **a**–**c** Scenarios 1–3. N1, N2, N1 + N2 represented effective population size of *O. longilobus*, *O. taihangensis*, and the common ancestor, respectively. Comparison of scenarios for direct estimate (**d**) and logistic regression (**e**) and both of them indicated that scenario 3 was the best support of these three scenarios. **f** The principal component analysis based on the posterior distributions for model checking in approximate Bayesian computation of the optimal scenarios 3

The PCA of the posterior distributions of model checking analysis revealed that the summary statistics from the observed data produced eigenvectors that were within, or at the margins of 1000 simulated pseudo-observed data sets (PODs), which indicated that scenario 3 was generally suitable for the observed data (Fig. 5). For Scenario 3, the low values of type I (direct estimate: 0.182; logistic estimate: 0.210) and type II errors (direct estimate: 0.094; logistic estimate: 0.093) were obtained based on 500 PODs. Under this best supported model, the estimated effective population sizes of *O. longilobus* and *O. taihangensis* were 2.21E + 04 (95% CI 4.22E + 03–1.24e + 05) and 7.40E + 04 (95% CI 1.36E + 04–3.51E + 05).

The historical gene flow generated using MIGRATE were low between the two species, *N*m _*O. longilobus* → *O. taihangensis*_ = 0.3813 (95% CIs 0–1.8790) and *N*m _*O. taihangensis* → *O. longilobus*_ = 0.7860 (95% CIs 0–3.1748). The mean contemporary gene flow (m) between the two species was also low. Migration occurred from *O. longilobus* to *O. taihangensis* and in turn was 0.0059 (95% CIs 0–0.0173) and 0.0052 (95% CIs 0–0.0156), respectively. However, the migration rates within each species were relatively high (*O. longilobus*: 0.9941 (95% Cis 0.9827–1.0055), *O. taihangensis*: 0.9948 (95% CIs 0.9844–1.0052)) as estimated by BAYESASS.

### Influences of environmental heterogenicity on *Opisthopappus* populations

**Fig. 6:**
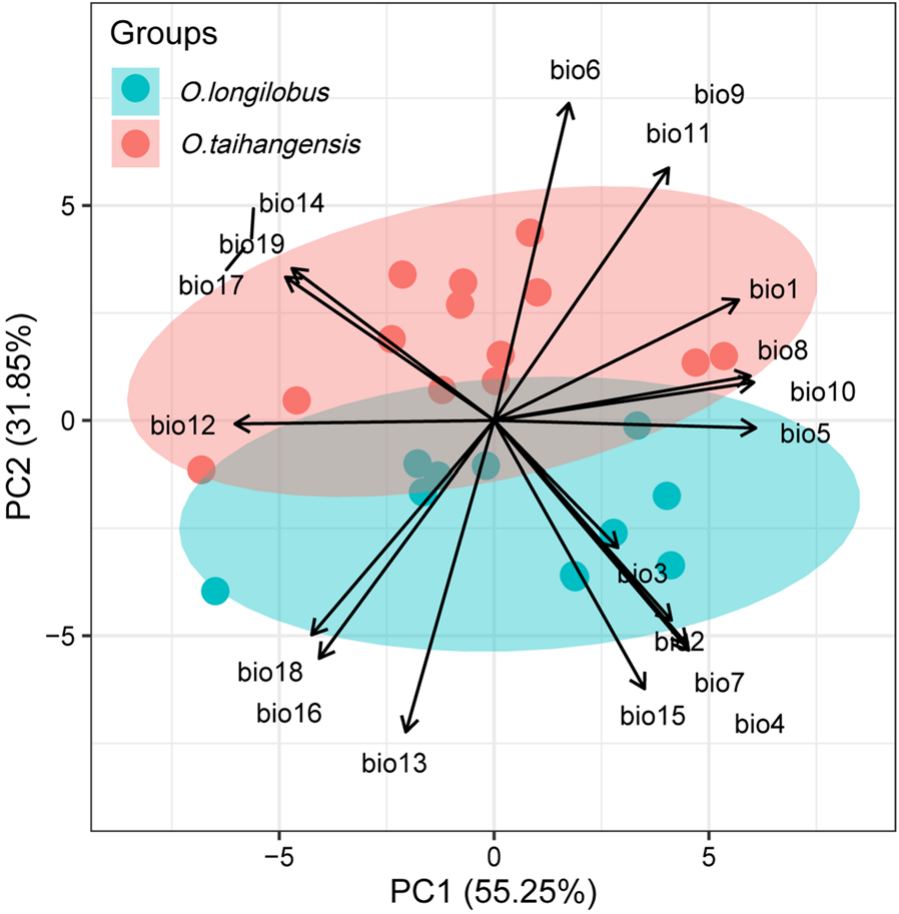
Principal component analysis of the climate factors of *O. longilobus* and *O. taihangensis.* The ellipses represent the 95% confidence interval of the distribution ranges of the sampling sites along PC1 and PC2

One-way ANOVA following the extraction of bioclimatic variables found that most of them, distributed along the two species were significantly different, including Mean diurnal range (bio2), Isothermality (bio3), Temperature seasonality (bio4), Min temperature of coldest month (bio6), Temperature annual range (bio7), Mean temperature of driest quarter (bio9), Mean temperature of coldest quarter (bio11), Precipitation of wettest month (bio13), Precipitation of driest month (bio14), Precipitation seasonality (bio15), Precipitation of wettest quarter (bio16), Precipitation of driest quarter (bio17), and Precipitation of coldest quarter (bio19) (Additional file 6: Table S2). The partial correlation of bioclimatic variables via PCA revealed that the explanatory direction was different for these variables. The PCA plot drawn on the first two axes explained 55.25% and 31.85% of the variations in the climate variables, respectively (Fig. 6).

To assess whether geographic or environmental differences might drive genetic differentiation, Barrier analysis, Mantel and partial Mantel tests were conducted. Among the *Opisthopappus* populations, geographical barriers existed (Fig. 1). In particular, more significant barriers were found between the boundary populations of *O. longilobus* and *O. taihangensis* (Fig. 1), which reflected a pattern of geographical isolation.

Significant associations between geographical and environmental distances appeared (r = 0.3588, P = 0.002) across all populations of *Opisthopappus*. Moreover, significant correlations were discovered in both the genetic and geographic distances (r = 0.5039, P = 0.001), and genetic and environmental distances (r = 0.3132, P = 0.001). The partial Mantel tests also detected significant correlations between genetic and environmental distances conditioned on geographic effects (r = 0.1631, P = 0.011).

Similar results, which revealed that geographic and environmental distances influenced genetic distances, were obtained by multiple matrix regression with randomization (MMRR) analyses. The effects were that both the geographic (coefficient = 0.2189, r^2^ = 0.2944, P = 0.001) and environmental (coefficient = 0.2060, r^2^ = 0.0944, P = 0.001) distances significantly related to the genetic distance. The joint effects of both the geographical and environmental distances also significantly impacted the genetic distance (r^2^ = 0.3108, coefficient _geo_ = 0.1955, P = 0.001, coefficient _env_ = 0.0751, P = 0.011).

**Fig. 7:**
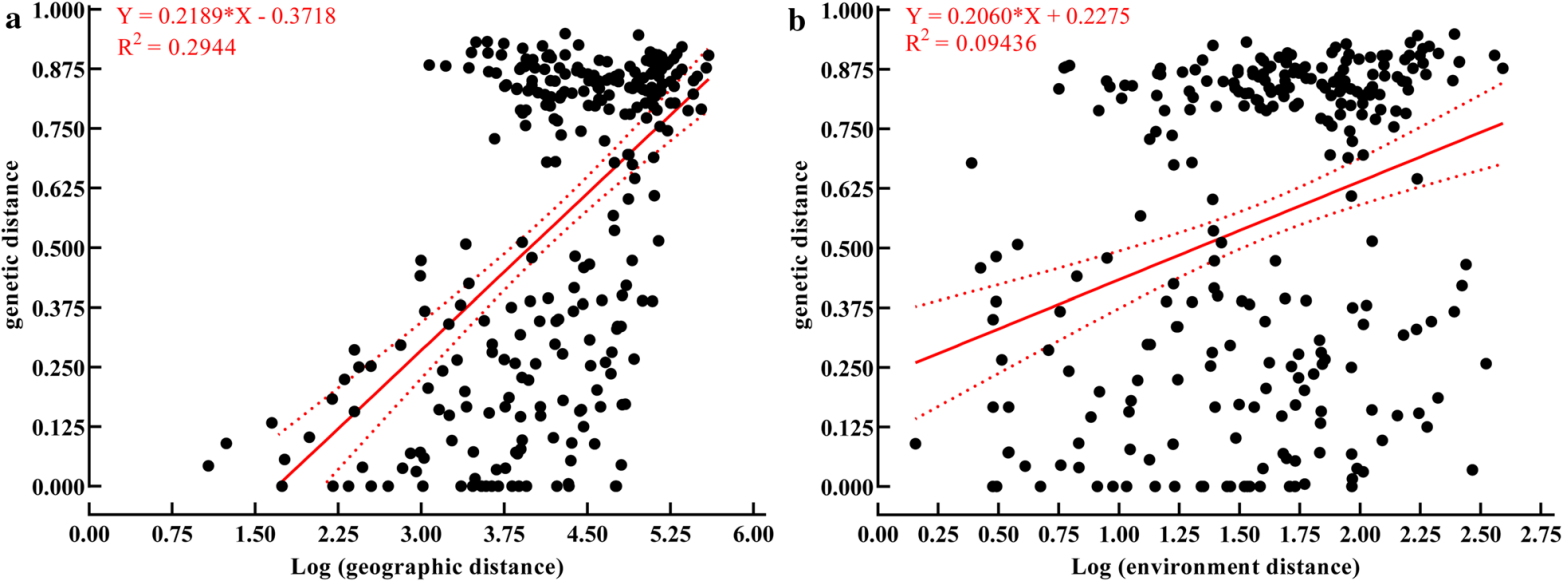
The scatterplots of the distribution of genetic distance along the geographic distance (**a**) and environment distance (**b**), respectively. The red line represents the multiple matrix regression with randomization (MMRR) equation with 95% confidence interval. **a** Geographic distance was positively correlated with genetic distance. MMRR: Slope = 0.2189 (95% CI 0.1786–0.2592), Y-intercept = − 0.3718 (95% CI − 0.5443–− 0.1992), X-intercept = 1.698 (95% CI 1.109–2.112), R^2^ = 0.2944, F = 114.3, P = 0.001. **b** Environment distance was also positively correlated with genetic distance. MMRR: Slope = 0.2060 (95% CI 0.1291–0.2829), Y-intercept = 0.2275 (95% CI 0.09839–0.3566), X-intercept = − 1.104 (95% CI − 2.734–− 0.3513), R^2^ = 0.09436, F = 27.82, P = 0.001

The above results indicated that the genetic differentiation of populations across the two species was significantly and linearly correlated with geographic and/or climatic differentiation. Scatterplots were subsequently constructed to show further details of the relationships between the genetic, geographical, and environmental distances (Fig. 7).

The full RDA model, including geographic distribution and climatic factors, explained 72.42% (conditioned: 12.22%, constrained: 60.20%, Table 3) of the variations between the genetic components. The partial RDA, while conditioned on the geographic distribution (coordinates) of sites, found a significant climate variable effect following the removal of the isolation by distance effects (Proportion = 60.20%, adj R^2^ = 0.5497, P = 0.03). The ANOVA indicated that the PC2 of bioclimatic variables significantly explained the genetic components with the highest explanatory proportions (Proportion = 56.75%, adj R^2^ = 0.5451, P = 0.01).

**Table 3:** Summary of partial dbRDA, showing the significance of climatic PCs (constrained factors) for explaining the variation in the genetic components

					GLM for the distribution of PCs along ordination axes						
	Inertia	Proportion	adjR^2^	P	t(axis1)	Pr( >|t|)	t(axis2)	Pr( >|t|)	Adj R^2^	F	P
Conditioned (Latitude + Longitude)	0.3496	12.22%	0.1053	0.0200							
Constrained	1.7223	60.20%	0.5497	0.0300							
PC1	0.0801	2.80%	0.0046	0.2300	− 0.7820	0.4430	1.4410	0.1640	− 0.0275	0.3840	0.5418
PC2	1.6236	56.75%	0.5451	0.0100	6.1110	0.0001	0.1740	0.8630	0.1687	5.6680	0.02636
PC3	0.0183	0.64%	0.0001	0.2900	0.1770	0.8610	2.7180	0.0126	0.1171	4.0500	0.0456
Unconstrained	0.7888	27.58%									
Total	2.8607	100.00%									

The distribution of the PC1-3 of climatic variables along the ordination axis was further examined by GLM (Table 4). The PC2 (adj R^2^ = 0.1687, F = 5.6682, P = 0.0264) and PC3 (adj R^2^ = 0.1171, F = 4.0500, P = 0.0456), had significant F statistics. PC2 correlated significantly with the ordination axis1 of dbRDA, while PC3 was significantly correlated with axis2. The high adjusted R^2^ indicated that PC2 and PC3 were sufficiently explained by the two dbRDA axes. Consistent influence estimates were obtained even when the order of predictors was altered; thus, the two variables were highly relevant for explaining the genetic differentiation of the populations across the two species. However, the adjusted R^2^ of PC1 might be too small to be meaningful in either dbRDA or GLM.

**Fig. 8:**
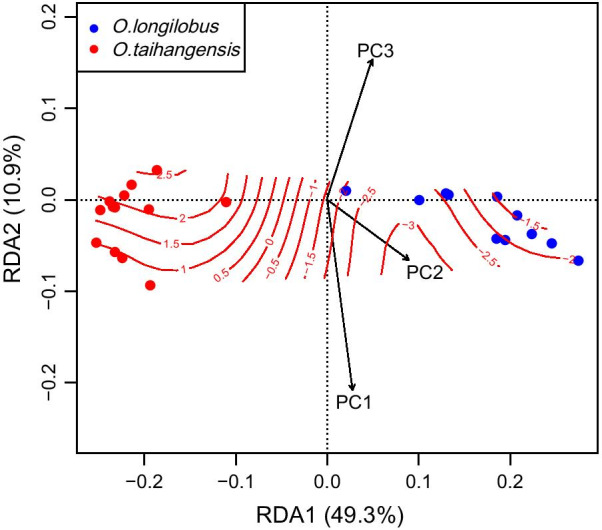
Scatter and ordisurf plots of the dbRDA for the PC2 of bioclimatic variables

Owing to its high explanatory proportions, the scatter and ordisurf plots (Fig. 8) for PC2 were further developed. The results congruently revealed broader ranges of environmental contours in *O. taihangensis* populations in contrast to *O. longilobus* populations. The populations of the two species demonstrated a significantly different distribution of the dbRDA space along axis 1, but not axis 2, which were similar to the DAPC clustering patterns. The distribution of populations within species was too close to be distinguished. The ordisurf plots clearly illustrated the climatic differentiation between *O. longilobus* and *O. taihangensis*.

## Discussion

### Genetic differentiation in localized heterogeneous environments of the Taihang Mountains

Abundant genetic variations were observed in *O. longilobus* or *O. taihangensis* (Table 1), which was consistent with previous researches [36–38]. As an ancestor of *O. taihangensis*, *O. longilobus* had a relatively higher genetic diversity than *O. taihangensis* (Table 1, Fig. 5) [42]. The perenniality and insect-pollination of these two species likely led to high genetic diversity, enabling them to persist across a range of environmental conditions on the Taihang Mountains.

The studied *Opisthopappus* populations could be well split into *O. longilobus* and *O. taihangensis* (Fig. 2–3)*,* which was slightly inconsistent with the previous results [36–38]. This might be from different molecular markers used. The markers used in the previous researches might reveal less genetic information than the markers used in this study that obtained across the whole genome of *Opisthopappus*. The EST-SSR markers developed from the transcriptome data also divided *Opisthopappus* populations into two clusters [43].

Overall genetic variations existed primarily among populations within *Opisthopappus* species (Table 2, Fig. 2–3, Additional file 3: Fig. S1-4). The genetic differentiation among populations or between species might be an outcome contributed to by both geography and environment (Figs. 1 and 7) [44–47].

With peaks reaching ~ 2800 m in elevation, the Taihang Mountains have many deep gullies and immense valleys. Its complex topography serves as a significant geographical barrier in Northern China [22], which can cause distributional gaps between species, leading not only to disjointed populations but also to cascading effects associated with interrupted gene flow and habitat heterogeneity [48]. Following limited gene exchange, *O. taihangensis* and *O. longilobus*, which are distributed only on the Taihang Mountains, were segmented into spatially isolated subpopulations (Figs. 1 and 7). Contemporary gene flow between the two species was found to be fairly low, and migration occurred primarily within the species. With the decline in gene flow, population differentiation would increase due to genetic drift or/and local adaptation effects under the conditions of heterogeneous habitats [47, 49].

From north to south, the weather of the Taihang Mountains changes from a temperate continental monsoon climate to a warm temperate semi-humid climate [21, 25]. The habitat for each population of *Opisthopappus* was diversified due to variable localized climates, such as temperature and precipitation.

Commonly, temperature and precipitation were found to play prominent roles as selective drivers for the variations in various plant species [17, 50–52]. Significant temperature fluctuations could contribute to the physiological states, metabolic levels, and genetic alterations of plants, to further drive the genetic makeup of populations. Meanwhile, precipitation during seed germination and growth would impact the demographic size as well as influencing successful seed colonization.

According our field investigations, various sample sites had different climatic conditions [24]. These differences in localized climates lead to the environmental isolation among *Opisthopappus* populations (Fig. 7). A driver of this pattern may be attributed to a neutral process of temporally disrupted gene flow between individuals growing in environmentally distinct habitats [53, 54]. In turn, these environmentally distinct habitats can serve as a barrier to gene flow, causing genetic differentiation between spatially close populations [47]. Consequently, these would promote isolated subpopulations to eventually evolve into genetically distinct lineages, while adapting to local environments [16, 17, 55].

In the Taihang Mountains, all extracted climatic variables might well be employed to explore weather differentiations between *O. taihangensis* and *O. longilobus* (Fig. 6, Additional file 6: Table S2). The PC2 of bioclimatic variables possessed an overwhelming explanatory ability toward genetic differentiation (Table 3, Fig. 8). Thereinto, bio6 and bio13 contained the first two longest projections with the rotation values in PC2 (Fig. 6). Bio6, the minimum temperature of the coldest month belonged to the temperature dimension, while bio13, the precipitation of the wettest month, belonged to the precipitation dimension.

Single or simple environmental variations could typically initiate the adaptive divergence between populations, followed by the expansion of accumulating genetic differentiation [5, 50, 56]. The significantly distinct distribution conditions and patterns of the two climate variables in the Taihang Mountains, corresponding to *O. longilobus* and *O. taihangensis*, might be regarded as original primordial indicators with driving forces toward causing and promoting the genetic differentiation and diversification of the two species.

### Evolutionary demographic dynamics from climatic transformation and topographic events

The most recent common ancestor of Asteraceae originated 76–66 Ma ago [57]. According to the fossil record, the Asteraceae family occurred at the Eocene–Oligocene boundary (42–49 Ma) [58, 59]. Zhao [60] pointed out that the Anthemideae tribe of Asteraceae originated in Eurasia, which then gradually dispersed eastward toward Asia. Some initial taxa of the Artemisiinae subtribe in central Asia evolved separately into *Dendranthema* and *Artemisia* groups. Within the *Artemisia* group, the oldest fossil pollen of the *Artemisia* genus was recorded at Eocene–Oligocene boundary [61, 62]. In the *Dendranthema* group, *Opisthopappus* is a relatively close taxon with *Ajania*, being the primary ancestral genus [60]. In our study, the temporal divergence between *Opisthopappus* and the outgroup *Chrysanthemum indicum* occurred at the Oligocene–Miocene boundary (25.08 Ma, 95% HPD: 22.14–27.92 Ma, Fig. 4). This period was coincidental with the evolution of the *Dendranthema* group.

Due to the collision of the India Plate with Eurasia, the Qinghai-Tibetan Plateau (QTP) began to lift during the Eocene (50–55 Ma) and then experienced different stages of growth to attain its current elevation [63–70]. The formation of the QTP dramatically modified the global and East Asian climate [70, 71] and triggered the Asian monsoon that had a significant impact on the weather in China [64]. The climatic systems of China were transformed from the original planetary to the monsoon system during the early Miocene, which created profound ecological heterogeneity [72]. It may be that this ecological heterogeneity drove the divergence of *Opisthopappus* from Asteraceae.

In the Burdigalian of Miocene (17.44 Ma, 95% HPD: 15.58–19.43 Ma), the *Opisthopappus* genus began to differentiate from *O. longilobus* to *O. taihangensis* (Fig. 4), which coincided with the second progressive and heterogeneous uplift of the QTP (15–13 Ma) [72–74]. During this period, the Asian monsoon was intensified owing to the extensive uplifting of the QTP. The monsoon characteristics of different areas resulted in the segregation of species populations [75].

During the early mid-Miocene period, the Asian monsoon was further enhanced, while global cooling events occurred in the ambient ocean and atmosphere [76]. However, following cooling events, the global temperature rapidly returned and the Middle Miocene Climate Optimum period (MMCO) emerged (14.7–16.9 Ma) [77]. Subsequently, as the Antarctic ice sheet expanded, the global weather changed from MMCO to a colder period. These climatic shifts served as a stimulus to promote the divergence of different plant populations [78]. Under this scenario, *O. longilobus* or *O. taihangensis* began to undergo intraspecific differentiation (Fig. 4), from 14.3 to 11.0 Ma during the Serravallian of Miocene periods.

Later in the Miocene (5.30–11.0 Ma) period, the continuously cooled global climate and the progressive extended QTP uplift brought the topographic and vegetational changes in China [79, 80]. In Eastern China, different weather systems (e.g., tropical humid, subtropical humid, warm temperate humid, and temperate humid) were gradually established from south to north [64]. The uplift process coupled with climatic changes initiated habitat diversification as well as that of the two species of *Opisthopappus*. This was verified by not only the divergence period of different intraspecific haplotypes (8.42–11.22 Ma), but the diverse haplotypes in each species (Fig. 4).

Toward the emergence of the Taihang Mountains the stage was being set for a neotectonics movement, from the late Miocene to early Pliocene [81]. The intermittent activity of the QTP, from 3.5 to 1.6 Ma (late Miocene to Pleistocene periods), drove the rapid uplift of Taihang Mountains during the Pleistocene. And the Taihang Mountain regions were within the ranges of monsoonal system at that time [82, 83]. The monsoon system interacted with the interglacial cycle to produce a more variable monsoon climate during the Pleistocene period [70, 83, 84]. The habitats of *O. longilobus* and *O. taihangensis* became ever more fragmented, where originally large and continuous populations may be separated into multiple smaller subpopulations. Thus, the most recent diversification of haplotypes within species occurred during the Pleistocene of the Quaternary [84], which also implied divergent selection between environments and localized adaptations to their respective habitats (Figs. 1 and 4).

Additionally, both *O. taihangensis* and *O. longilobus* exhibited signs of recent expansion during the evolutionary process (Additional file 5: Table S1). This was confirmed by the distribution of the *Opisthopappus* haplotypes (Fig. 1) and network (Additional file 4: Fig. S4). During the Quaternary period, the paleovegetation of Taihang Mountains repeatedly appeared as replacement species during grassland and forest cycles [85]. Emerging grasslands might have served as a transitional corridor that provided opportunities for populations to expand and colonize.

### Implication of the outcome from this research

Spatial environmental heterogeneity is typically proposed as a critical driver that leads to population differentiation, and even the acceleration of speciation. Here, we provided comprehensive evidence, including genetics, geographical conditions, climate variables, and evolutionary processes to interpret the differentiation of two *Opisthopappus* species.

Based on the above results, the divergence and intraspecies variations of *Opisthopappus* primarily resulted from climate fluctuations, the intensification of Asian monsoon, and the topographic complexity of China with the extensive uplift of the QTP. Subsequently, the ecological stratification and environmental heterogeneity of different climatic systems and the rapid rise of the Taihang Mountains shaped the contemporary geographical distribution pattern of the two *Opisthopappus* species.

Our results indicated that ecological factors play important role in shaping the physiological states, metabolic levels, and genetic alterations of species and populations and might drive the genetic makeup of populations and species. The results provide useful information for us to understand the ecology and evolution of organisms in the mountainous environment from population and species perspective.

## Conclusion

In summary, when genetics, geographical conditions, climate variables, and evolutionary processes were all considered, *O. taihangensis* and *O. longilobus* were clearly distinct. At ~ 17.44 Ma during the early Miocene, the establishment of differing monsoon regimes due to the enhanced Asian monsoon from the QTP uplift triggered the derivation of *O. taihangensis* from *O. longilobus*. During the mid- late Miocene period, dramatic climatic shifts coupled with the progressive and heterogeneous uplift of the QTP initiated the intraspecific differentiation of these two species. Up until the Pleistocene, the rapid uplift of the Taihang Mountains coupled with violent climatic oscillations further promoted the diversity of the two species. With the formation of the Taihang Mountains, this complex topography led to localized environments and ecological heterogeneity, which established spatiotemporal isolation between populations. Under this scenario, *O. taihangensis* and *O. longilobus* underwent adaptive divergence, which gradually shaped current genetic structures and distribution patterns. The results of this study explored the differentiation mechanisms of these two species of the *Opisthopappus* genus, revealing the impacts of environmental events by taking small-scale spatial niches into consideration, while providing clues for the further investigation of other germplasm resources of the Taihang Mountains.

## Methods

### Sample collection

Our study was conducted in accordance with the laws of the People’s Republic of China, and field collection was approved by the Chinese Government. All researchers received permission letters from the College of Life Science, Shanxi Normal University, to collect the samples, which were taxonomically identified based on their phenotype by Junxia Su (Associate Professor of systematic botany) at Shanxi Normal University. The voucher specimens were deposited in the herbarium of College of Life Science, Shanxi Normal University (No:20170105030–20170105050).

Eleven populations of *O. longilobus* and thirteen populations of *O. taihangensis* were sampled, which covered the *Opisthopappus* distribution ranges (Table 1, Fig. 1). Individuals growing at a common site were regarded as a single "population". Fresh young leaves devoid of disease or insect pests were selected for each of the sample sites, where 10–15 individuals from each population were collected. These samples were placed into sealed bags filled with silica gel, dehydrated/quickly dried, and stored at 20 °C for later use. A global positioning system (GPS) was employed to demarcate each sample site and record the longitude, latitude, and elevation of each population (Table 1).

### PCR amplification, sequencing, and genotyping

The total genomic DNA was extracted using the modified 2 × CTAB method [71]. The quality of DNA was measured using an ultraviolet spectrophotometer and 0.8% agarose gel electrophoresis, and stored at − 20 °C for further use.

The SNP and InDel primers (Additional file 8: Table S3) of nuclear genes of *Opisthopappus* were obtained from a pervious study [41]. For the SNP primers, the 20 µL PCR reaction contained 10 µL 2 × MasterMix, 2 µL template DNA (30 ng/µL), 1 µL primer S (10 µM), 1 µL primer A (10 µM), and 6 µL ddH_2_O. The PCR procedure proceeded as follows: pre-denaturation at 94 °C for 5 min., denaturation at 94 °C for 1 min, annealing temperature based on each primer setting for 1 min, elongation at 72 °C for 1.5 min., repeated for 35 cycles, last elongation at 72 °C for 10 min, and preservation at 4 °C. The PCR products detected using 2% agarose gel electrophoresis were confirmed via an automatic analysis electrophoresis gel imaging system, which were then sent to Sangon Biotech (Shanghai) for sequencing.

For the InDel primers, the PCR reaction was 20 µL, which contained 10 µL 2 × MasterMix, 3 µL template DNA (30 ng/µL), 1 µL primer S (10 µM), 1 µL primer A (10 µM), and 5 µL ddH_2_O. The PCR procedure was as follows: pre-denaturation at 94 °C for 1 min, denaturation at 94 °C for 1 min, annealing temperature based on each primer setting for 1 min, elongation at 72 °C for 1 min, repeated for 35 cycles, last elongation at 72 °C for 10 min, preservation at 4 °C. The PCR products were detected using 8% polyacrylamide gel electrophoresis. The presence or absence of each InDel fragment were coded as ‘1′and ‘0′ respectively. The details for the numbers of individuals for SNP sequencing and InDel genotyping are shown in Table 1.

### Population genetic differentiation analyses

Prior to population genetic analysis, the partition homogeneity test (PHT) were initially conducted by PAUP [86] to identify whether the SNP sequences were suitable to be combined. The non-significant (P > 0.05) of the results revealed that the combined SNP sequences were suitable.

The haplotypes, haplotype frequencies, haplotype diversity (*H*d), and nucleotide diversity (π) were calculated using DNASP 5.10 [87]. The genetic *G*_ST_ and *N*_ST_ differentiation parameters were examined by PERMUT 2.0 [88] based on the haplotype frequency.

For the InDel data, the genetic characteristics, Nei's gene diversity index (*H*), Shannon’s information index (*I*), and the percentage of polymorphic loci (*PPL*), were calculated by POPGENE 1.31 [89]. An analysis of molecular variance (AMOVA) was implemented by ARLEQUIN 3.5 [90] and GENALEX 6.5 [91] to detect the distribution of genetic variations within and between populations or species. Subsequently, the *F*_ST_, *F*_CT_, and *F*_SC_ values [92] were calculated based on hierarchical AMOVA, and the permutation test was set to 1000.

Cluster analysis based on the maximum likelihood (ML) method and Nei’s genetic distance, respectively, was performed using MEGA 7.0 [93]. Bayesian clustering analysis (BCA) was employed to examine the similarity and divergence of genetic components between populations and performed using STRUCTURE 2.2 [94] for both the SNP sequencing and InDel data. The posterior probability of grouping number (*K* = 2–24) was estimated through 10 independent runs using 500,000 step Markov chain Monte Carlo (MCMC) replicates, following a 1,000,000-step burn-in for each run to evaluate consistency. The best grouping number was evaluated by Δ*K* [95] in STRUCTURE HARVESTER 0.6.94 [96]. These 10 runs were aligned and summarized using CLUMPP 1.1.2 [97] and the visualization of the results was plotted using DISTRUCT 1.1 [98].

To test the genetic differentiation between populations or species, a discriminant analysis of principal components (DAPC) was implemented by the function dapc in the R package ‘adegenet’ [99], which initially transformed the genetic data using principal component analysis (PCA) results, and subsequently performed discriminant analysis on the retained principal components. The properties of the “without a priori”, using partial synthetic variables to minimize variations within groups [100], might assist with objectively evaluating the artificial classification of *O. taihangensis* and *O. longilobus*. Kruskal–Wallis tests for the first two principal components (PCs), and the first two linear discriminants (LDs) of DAPC, were conducted to examine the genetic differentiation between the populations and species.

### Inference of population demographic history

A network relationship was generated through the median-joining method in POPART 1.7 [101], to investigate the evolutionary relationships between the *Opisthopappus* haplotypes. BEAST 1.84 [102] was employed to estimate the differentiation and diversification time between haplotypes. *Chrysanthemum indicum,* belonging to the same subtribe of Chrysantheminae with *Opisthopappus* (holding identified genomic information) was selected as the outgroup in BEAST analysis. The haplotype sequence of each primer was aligned to the NT (Nucleotide Sequence) database followed by manual splicing. Owing to the absence of the record of the *Opisthopappus* fossil data, the divergence time of *Chrysanthemum* and *Opisthopappus* (25.40 Ma) referred to the Time Tree website (http://www.timetree.org/) was adopted as a prerequisite for calibrating the age of most recent common ancestor (tMRCA).

The Akaike Information Criterion (AIC) with a “greedy” algorithm in PartitionFinder 2.1.1 [103] was employed to select the best-fit partitioning schemes and evolutionary models. Based on the AIC results, the dataset was partitioned into four groups (group1: SNP2 + SNP29, group2: SNP4 + SNP26, group3: SNP13 + SNP32, and group4: SNP19 + SNP23), and the phylogenetic relationships were inferred based on four optimal evolutionary models, namely HKY + I + G + X, HKY + I + G, SYM + I + G and GTR + I + X, corresponding to group1 to group4, respectively. The generic average mutation rate of 6.1 × 10^–9^ (5.1 and 7.1 × 10^–9^) for the nuclear DNA of the Asteraceae species was employed according to the present study [75]. Markov chain Monte Carlo (MCMC) was repeated 8 × 10^7^ times by sampling every 80,000 generations. TRACER 1.5 [102] was used to check the convergence of the framework, which ensured that every tested parameter was greater than 200.

To assess whether the species had experienced a significant expansion, we utilized ARLEQUIN 3.5 [90] to calculate the Tajima’s *D* [104] and Fu’s *F*_S_ [105] values. Moreover, the sum of square deviation (SSD) and raggedness index (Rag) in the mismatch distribution analysis (MDA) was performed in ARLEQUIN 3.5. The process employed a 1000 step permutation test.

Approximate Bayesian computation (ABC) analysis, provided by DIY-ABC 2.1.0 [106], enabled the estimation of complex evolutionary population histories. Based on the estimated genetic variations, genetic structures, and current geographic distributions, three evolutionary scenarios were proposed. Scenario 1: *O. longilobus* and *O. taihangensis* were differentiated from a common ancestral population during the same period. Scenario 2: *O. taihangensis* was an ancestral population, and *O. longilobus* was differentiated from *O. taihangensis*. Scenario 3: *O. longilobus* was the ancestral population, and *O. taihangensis* was differentiated from *O. longilobus*.

Each scenario was performed with 1,000,000 simulations and six summary statistics (number of haplotypes, number of segregating sites, mean of pairwise differences, Tajima’s D and private segregating sites) were selected. The substitution rates of nuclear genes were the same as those used in the BEAST analysis. To identify the best-supported scenario under direct and logistic approaches, we selected 1% of the simulated datasets closest to the observed data to evaluate model accuracy and estimate the relative posterior probability (PP) with 95% confidence intervals (95% CI) for each scenario. Further, the parameters including effective population size and divergence generation was estimated under the optimal scenario. The goodness of fit of the best supported scenario was evaluated by the option ‘model checking’ with principal component analysis (PCA). To estimate type I and II errors on the power of model selection, we assessed confidence in scenario choice with 500 simulated pseudo-observed data sets (PODs) for the seven plausible scenarios.

Additionally, the historical and contemporary gene flow were estimated within the two *Opisthopappus* species by MIGRATE-N 3.6 [107] and BAYESASS 3.0 [108], respectively. In MIGRATE-N 3.6, maximum-likelihood analyses were performed using 10 short chains (10^4^ trees) and three long chains (10^5^ trees) with 10^4^ trees discarded as an initial burn-in’ and astatic heating scheme at four temperatures (1, 1.5, 3, and 1000,000). To ensure the consistency of estimates, we repeated this procedure five times and reported average maximum-likelihood estimates with 95% confidence intervals. The parameters θ and *M* were estimated using a Bayesian method, which could be employed to estimate the number of migrants per generation (*N*m) into each population using the Eq. 4*N*m = θ*M.

When estimating the contemporary gene flow using BAYESASS 3.0, the parameters were examined including migration rates (m), allele frequencies (a) and inbreeding coefficients (f) to ensure that the optimal acceptance rates of the three parameters fell within the 20–60% range. Ten independent runs were executed to minimize the convergence problem. The result with the lowest deviance was adopted according to the method of Meirmans [109], where the 95% credible interval was estimated as m ± 1.96 × standard deviation (SD).

### Environmental variables influence analyses

Nineteen bioclimatic variables (Bioclim) representing Grinnellian niches [110, 111], which are defined as the scenopoetic environmental variables of a species required to survive, were downloaded from the WorldClim database (http://www.worldclim.org/) with a resolution of 30 arc-sec (~ 1 × 1 km) and extracted using the R package ‘raster’ [112]. Subsequently, the significance test of the distribution of climate factors along the two species was tested by one-way ANOVA. A principal component analysis (PCA) of independent climatic variables to reduce the dimensionality that defined the niche space, allowed for the comparison of the integrity of climate variables between *O. longilobus* and *O. taihangensis*, after which the PC1–PC3 were reserved for further analysis.

To test how the geographical and environmental differences impacted genetic differentiation, the Mantel test, partial Mantel test, and Barrier analysis were applied in this study. Further, a multiple matrix regression with randomization (MMRR) was performed to explore whether the genetic distance responded to variations in geographic and/or environmental distances.

Pairwise *F*_ST_ distance calculated in ARLEQUIN 3.5 was used as the genetic distance. The geographic distance was estimated using the GENALEX 6.5 according to three-dimensional factors (latitude, longitude, and elevation). The environmental distance was calculated using the Euclidean distance with PASSAGE 2.0 based on the first three PCs [113].

The Mantel test was performed in the R package ‘vegan’ [114], whereas the MMRR analysis was performed using the R package ‘PopGenReport’ [115, 116]. Logarithmic transformation of the distance matrices was conducted to ensure that they are in the same or similar order of magnitude. Regression coefficients of the Mantel test (r) and MMRR (r^2^) and their significance were determined based on 9,999 random permutations. Scatterplots to reveal the relationships between genetic, environmental, and geographic distances were conducted using GraphPad Prism 8 [117].

The biogeographic boundaries between population pairs were calculated by the Monmonier’s maximum-difference algorithm in BARRIER 2.2 [118] based on the multiple distance matrix. Permutation and bootstrap tests were conducted with 1000 replicates for each case (Fig. 1).

In addition, distance based redundancy analyses (dbRDA) were performed to elucidate whether the climatic variables conditioned on the geographic distribution explained the genetic differentiation of the populations using the R package ‘vegan’. Firstly, a distance-based principal coordinate analysis (PCoA) of the genetic data at the species level was performed to generate several principal coordinates (PCs) using the R package ‘ape’ [119]. Next, the PC1-3 of climatic variables were employed as explanatory variables conditioned on geographic factors, and significance tests were performed using the “anova. cca” [120] function in the R package ‘vegan’ with 999 permutations. The distribution pattern of the PC1-3 of climate variables along the ordination axes1-2 was further analyzed using a generalized linear model (GLM). Finally, the first two RDA axes and the explanatory variables were employed to construct the ordination and ordisurf plots of the dbRDA.

## Supplementary Information

**Additional file 1: Fig. S1.** Phylogenetic relationships between *O. longilobus* and *O. taihangensis*. (A): Individual ML clustering of *Opisthopappus.* Blue branches presented individuals of *O. longilobu*s and red branches presented individuals of *O. taihangensis*. (B): UPGMA clustering for 24 populations of *Opisthopappus* based on Nei’s genetic distance. Blue for populations of *O. longilobus* and red for populations of *O. taihangensis*.

**Additional file 2: Fig. S2.** Structure analysis from *K* = 3 to *K* = 6 for SNP and InDel, respectively.

**Additional file 3: Fig. S3** The Kruskal–Wallis test of the first two principal components and the first two linear discriminants of the genetic variation revealed significant genetic divergence between species but no or little population differentiation within species. (A–D): Comparisons between species. (E–H): Comparisons among populations.

**Additional file 4: Fig. S4.** Haplotypes network of *Opisthopappus.* 47 haplotypes (H1-H47) were detected in *O. longilobus* and 28 haplotypes (H48-H75) in *O. taihangensis*. No shared haplotypes were detected between *O. longilobus* and *O. taihangensis*. The color of each haplotype corresponded to Fig. 1 The size of the circles corresponds to the frequency of each haplotype and each solid line represents one mutational step.

**Additional file 5: Table S1.** The results of neutrality tests (Tajima’s D and Fu’s FS tests) and mismatch distribution analyses.

**Additional file 6: Table S2.** ANOVA analysis for the nineteen bioclimatic variables grouped by two different species.

**Additional file 7: Fig. S5.** The average temperature of every month **(A)** and the average precipitation of every month **(B)** of the studied populations of the distribution of *Opisthopappus.*

**Additional file 8: Table S3.** Information of primer pairs.

## Data Availability

The datasets generated and/or analyzed during the current study was available in the Dryad repository, https://doi.org/10.5061/dryad.p5hqbzkpd. The datasets used and analyzed during the current study was also available from the corresponding author on reasonable request.

## Abbreviations

AMOVA: Analysis of molecular variance
BCA: Bayesian clustering analysis
PCA: Principal components analysis
PC: Principal components
LD: Linear discriminants
K-W test: Kruskal–Wallis test
MDA: Mismatch distribution analysis
ABC: Approximate Bayesian computation
MMRR: Multiple matrix regression with randomization
dbRDA: Distance based redundancy analysis
GLM: Generalized linear model
AIC: Akaike information criterion
