# Supplementary material for: Localized environmental heterogeneity drives the population differentiation of two endangered and endemic Opisthopappus Shih species
Source: BMC Ecol Evol. 2021 Apr 15;21:56. doi: 10.1186/s12862-021-01790-0 (PMC8050911; doi:10.1186/s12862-021-01790-0)
Supplement: Supplementary file 1 — Additional file 1: Fig. S1. Phylogenetic relationships between O. longilobus and O. taihangensis. (A): Individual ML clustering of Opisthopappus. Blue branches presented individuals of O. longilobus and red branches presented individuals of O. taihangensis. (B): UPGMA clustering for 24 populations of Opisthopappus based on Nei’s genetic distance. Blue for populations of O. longilobus and red for populations of O. taihangensis. [file 12862_2021_1790_MOESM1_ESM.docx]

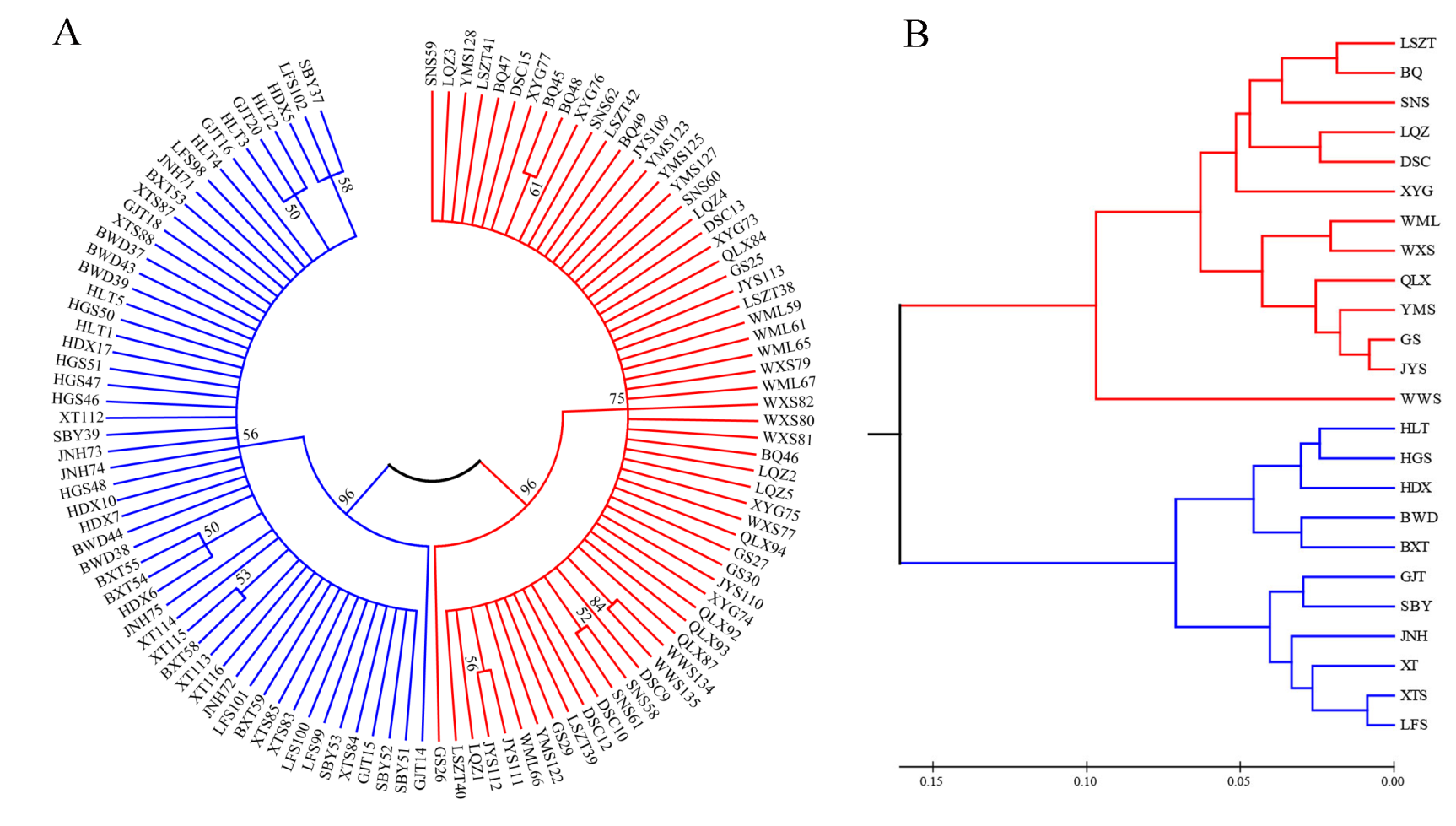


**Additional file 1: Fig. S1** Phylogenetic relationship between *O. longilobus* and *O. taihangensis*. (A): Individual ML clustering of *Opisthopappus.* Blue branches presented individuals of *O. longilobu*s and red branches presented individuals of *O. taihangensis*. (B): UPGMA clustering for 24 populations of *Opisthopappus* based on Nei’s genetic distance. Blue for populations of *O. longilobus* and red for populations of *O. taihangensis*.
