# Supplementary figures and images for: Localized environmental heterogeneity drives the population differentiation of two endangered and endemic Opisthopappus Shih species
Source: BMC Ecol Evol. 2021 Apr 15;21:56. doi: 10.1186/s12862-021-01790-0 (PMC8050911; doi:10.1186/s12862-021-01790-0)

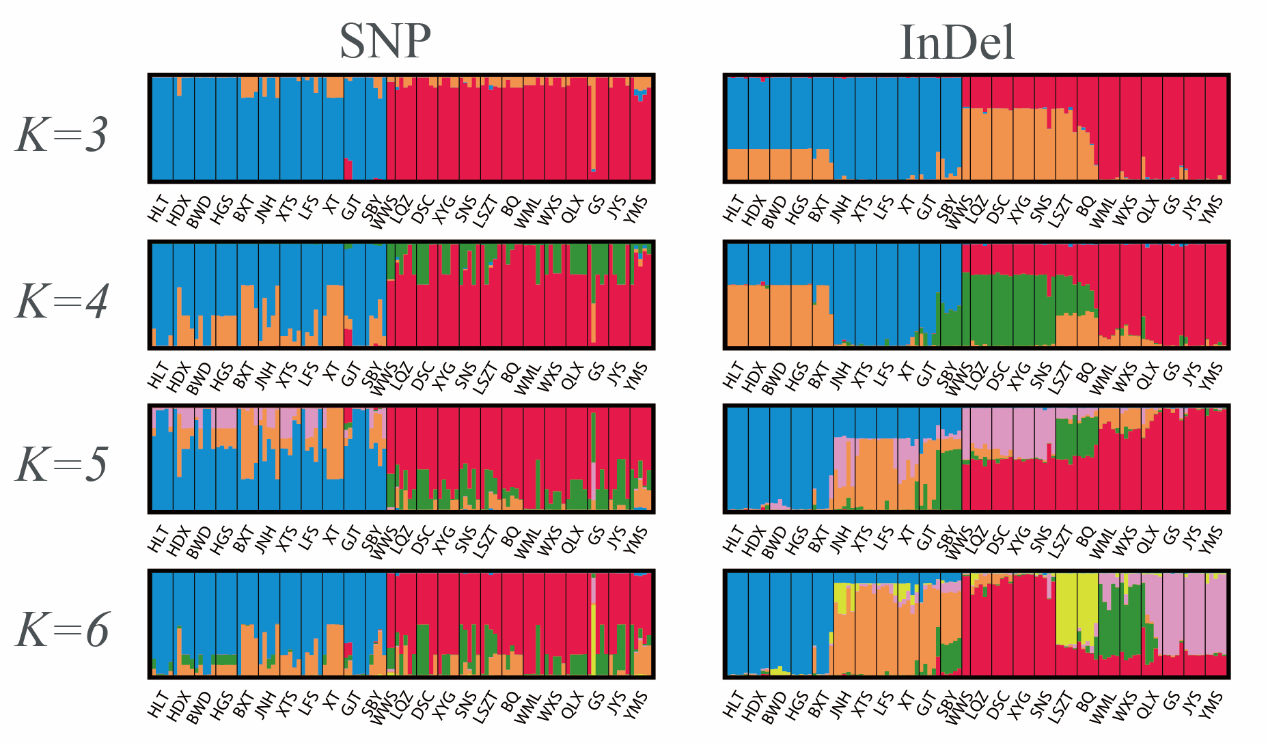


**Additional file 2: Fig. S2** Structure analysis from *K=3* to *K=6* for SNP and InDel, respectively.

Supplement: Supplementary file 2 — Additional file 2: Fig. S2. Structure analysis from K = 3 to K = 6 for SNP and InDel, respectively. [file 12862_2021_1790_MOESM2_ESM.docx]
