# Supplementary material for: Localized environmental heterogeneity drives the population differentiation of two endangered and endemic Opisthopappus Shih species
Source: BMC Ecol Evol. 2021 Apr 15;21:56. doi: 10.1186/s12862-021-01790-0 (PMC8050911; doi:10.1186/s12862-021-01790-0)
Supplement: Supplementary file 3 — Additional file 3: Fig. S3 The Kruskal–Wallis test of the first two principal components and the first two linear discriminants of the genetic variation revealed significant genetic divergence between species but no or little population differentiation within species. (A–D): Comparisons between species. (E–H): Comparisons among populations. [file 12862_2021_1790_MOESM3_ESM.docx]

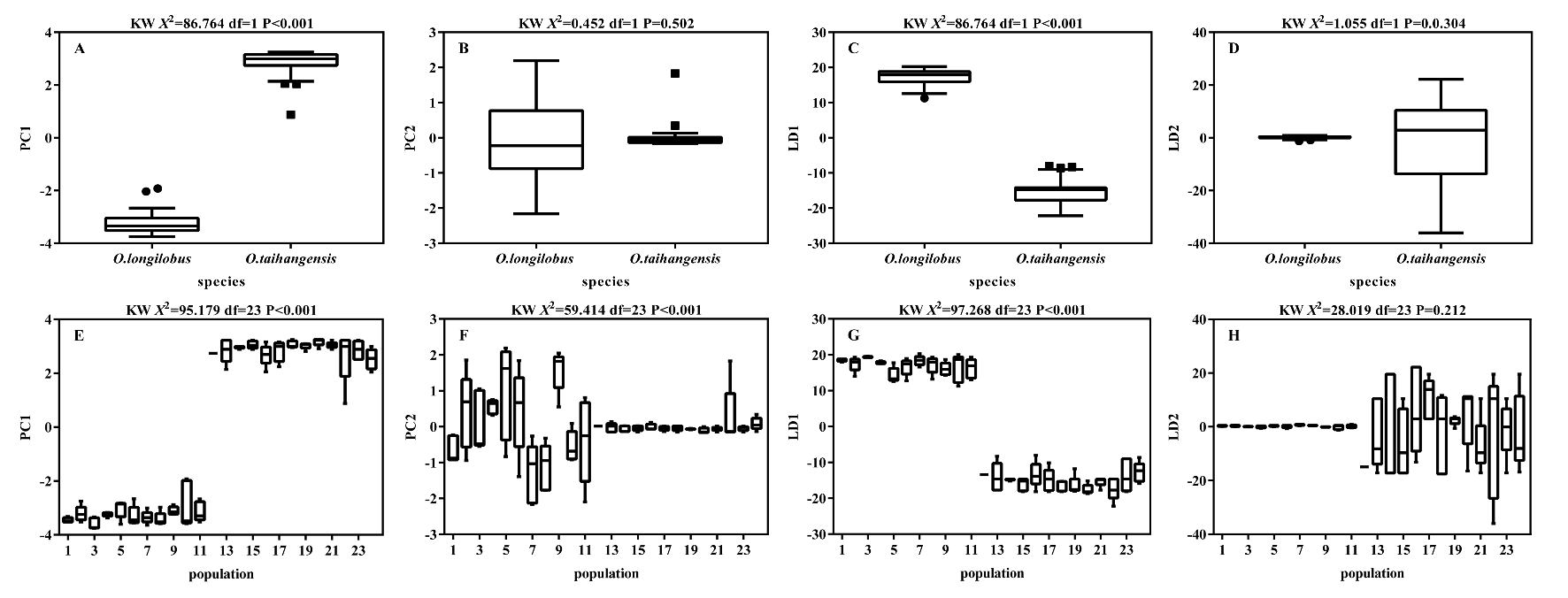


**Additional file 3: Fig. S3** The Kruskal–Wallis test of the first two principal components and the first two linear discriminants of the genetic variation revealed significant genetic divergence between species but no or little population differentiation within species. (A–D): Comparisons between species. (E–H): Comparisons among populations.
