# Supplementary material for: Localized environmental heterogeneity drives the population differentiation of two endangered and endemic Opisthopappus Shih species
Source: BMC Ecol Evol. 2021 Apr 15;21:56. doi: 10.1186/s12862-021-01790-0 (PMC8050911; doi:10.1186/s12862-021-01790-0)
Supplement: Supplementary file 4 — Additional file 4: Fig. S4. Haplotypes network of Opisthopappus. 47 haplotypes (H1-H47) were detected in O. longilobus and 28 haplotypes (H48-H75) in O. taihangensis. No shared haplotypes were detected between O. longilobus and O. taihangensis. The color of each haplotype corresponded to Fig. 1 The size of the circles corresponds to the frequency of each haplotype and each solid line represents one mutational step. [file 12862_2021_1790_MOESM4_ESM.docx]

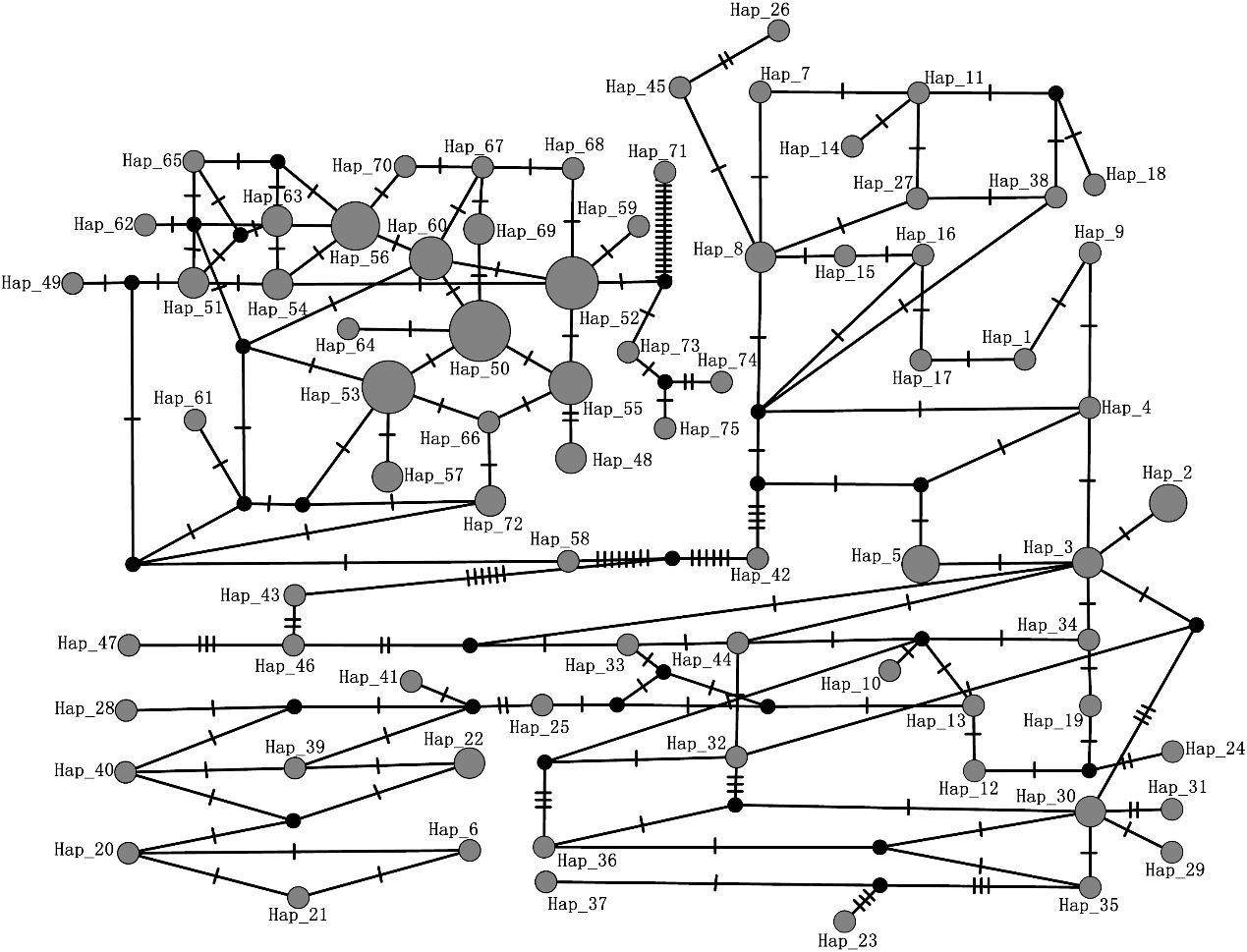


**Additional file 4: Fig. S4** Haplotypes network of *Opisthopappus.* 47 haplotypes (H1-H47) were detected in *O. longilobus* and 28 haplotypes (H48-H75) in *O. taihangensis*. No shared haplotypes were detected between *O. longilobus* and *O. taihangensis*. The size of the circles corresponds to the frequency of each haplotype and each solid line represents one mutational step.
