# Supplementary material for: Localized environmental heterogeneity drives the population differentiation of two endangered and endemic Opisthopappus Shih species
Source: BMC Ecol Evol. 2021 Apr 15;21:56. doi: 10.1186/s12862-021-01790-0 (PMC8050911; doi:10.1186/s12862-021-01790-0)
Supplement: Supplementary file 5 — Additional file 5: Table S1. The results of neutrality tests (Tajima’s D and Fu’s FS tests) and mismatch distribution analyses. [file 12862_2021_1790_MOESM5_ESM.docx]

| Additional file 5: Table S1. The results of neutrality tests (Tajima’s *D* and Fu’s *F_S_* tests)  and mismatch distribution analyses | | | | | | | | | | |
| --- | --- | --- | --- | --- | --- | --- | --- | --- | --- | --- |
|  | Neutrality tests | | | |  | Mismatch analyses | | | | |
|  | Tajima’s *D* | P value | Fu’s *F_S_* | P value |  | SSD | P value | Rag | P value |  |
| *O. longilobus* | 0.4058 | 0.7174 | -25.2963 | 0.01 |  | 0.0008 | 0.8100 | 0.0072 | 0.91 |  |
| *O. taihangensis* | -1.4357 | 0.0527 | -18.5566 | 0.01 |  | 0.0008 | 0.6600 | 0.0315 | 0.41 |  |
| *Opisthopappus* | 1.4589 | 0.8741 | -24.1000 | 0.01 |  | 0.0291 | 0.2500 | 0.0068 | 0.57 |  |
