# Supplementary material for: Localized environmental heterogeneity drives the population differentiation of two endangered and endemic Opisthopappus Shih species
Source: BMC Ecol Evol. 2021 Apr 15;21:56. doi: 10.1186/s12862-021-01790-0 (PMC8050911; doi:10.1186/s12862-021-01790-0)
Supplement: Supplementary file 6 — Additional file 6: Table S2. ANOVA analysis for the nineteen bioclimatic variables grouped by two different species. [file 12862_2021_1790_MOESM6_ESM.docx]

Additional file 6: Table S2 ANOVA analysis for the nineteen bioclimatic variables grouped by two different species

|  | | Sum of Squares | df | Mean Square | F | Sig. |
| --- | --- | --- | --- | --- | --- | --- |
| bio1 | Between Groups | 1.131 | 1 | 1.131 | .465 | .503 |
|  | Within Groups | 53.542 | 22 | 2.434 |  |  |
|  | Total | 54.673 | 23 |  |  |  |
| bio2 | Between Groups | 7.456 | 1 | 7.456 | 13.219 | .001 |
|  | Within Groups | 12.409 | 22 | .564 |  |  |
|  | Total | 19.865 | 23 |  |  |  |
| bio3 | Between Groups | 4.528 | 1 | 4.528 | 4.786 | .040 |
|  | Within Groups | 20.815 | 22 | .946 |  |  |
|  | Total | 25.344 | 23 |  |  |  |
| bio4 | Between Groups | 26779.925 | 1 | 26779.925 | 17.679 | .000 |
|  | Within Groups | 33325.715 | 22 | 1514.805 |  |  |
|  | Total | 60105.640 | 23 |  |  |  |
| bio5 | Between Groups | 3.152 | 1 | 3.152 | .791 | .383 |
|  | Within Groups | 87.662 | 22 | 3.985 |  |  |
|  | Total | 90.813 | 23 |  |  |  |
| bio6 | Between Groups | 25.945 | 1 | 25.945 | 25.135 | .000 |
|  | Within Groups | 22.709 | 22 | 1.032 |  |  |
|  | Total | 48.653 | 23 |  |  |  |
| bio7 | Between Groups | 47.181 | 1 | 47.181 | 18.080 | .000 |
|  | Within Groups | 57.412 | 22 | 2.610 |  |  |
|  | Total | 104.593 | 23 |  |  |  |
| bio8 | Between Groups | .144 | 1 | .144 | .051 | .823 |
|  | Within Groups | 62.174 | 22 | 2.826 |  |  |
|  | Total | 62.318 | 23 |  |  |  |
| bio9 | Between Groups | 11.624 | 1 | 11.624 | 7.356 | .013 |
|  | Within Groups | 34.765 | 22 | 1.580 |  |  |
|  | Total | 46.389 | 23 |  |  |  |
| bio10 | Between Groups | .356 | 1 | .356 | .097 | .759 |
|  | Within Groups | 80.842 | 22 | 3.675 |  |  |
|  | Total | 81.198 | 23 |  |  |  |
| bio11 | Between Groups | 11.624 | 1 | 11.624 | 7.356 | .013 |
|  | Within Groups | 34.765 | 22 | 1.580 |  |  |
|  | Total | 46.389 | 23 |  |  |  |
| bio12 | Between Groups | 729.232 | 1 | 729.232 | .654 | .427 |
|  | Within Groups | 24512.601 | 22 | 1114.209 |  |  |
|  | Total | 25241.833 | 23 |  |  |  |
| bio13 | Between Groups | 801.947 | 1 | 801.947 | 15.957 | .001 |
|  | Within Groups | 1105.678 | 22 | 50.258 |  |  |
|  | Total | 1907.625 | 23 |  |  |  |
| bio14 | Between Groups | 5.549 | 1 | 5.549 | 5.827 | .025 |
|  | Within Groups | 20.951 | 22 | .952 |  |  |
|  | Total | 26.500 | 23 |  |  |  |
| bio15 | Between Groups | 518.263 | 1 | 518.263 | 22.624 | .000 |
|  | Within Groups | 503.960 | 22 | 22.907 |  |  |
|  | Total | 1022.223 | 23 |  |  |  |
| bio16 | Between Groups | 1338.126 | 1 | 1338.126 | 6.068 | .022 |
|  | Within Groups | 4851.832 | 22 | 220.538 |  |  |
|  | Total | 6189.958 | 23 |  |  |  |
| bio17 | Between Groups | 67.975 | 1 | 67.975 | 6.747 | .016 |
|  | Within Groups | 221.650 | 22 | 10.075 |  |  |
|  | Total | 289.625 | 23 |  |  |  |
| bio18 | Between Groups | 785.596 | 1 | 785.596 | 2.555 | .124 |
|  | Within Groups | 6764.238 | 22 | 307.465 |  |  |
|  | Total | 7549.833 | 23 |  |  |  |
| bio19 | Between Groups | 67.975 | 1 | 67.975 | 6.747 | .016 |
|  | Within Groups | 221.650 | 22 | 10.075 |  |  |
|  | Total | 289.625 | 23 |  |  |  |
