# Supplementary material for: Localized environmental heterogeneity drives the population differentiation of two endangered and endemic Opisthopappus Shih species
Source: BMC Ecol Evol. 2021 Apr 15;21:56. doi: 10.1186/s12862-021-01790-0 (PMC8050911; doi:10.1186/s12862-021-01790-0)
Supplement: Supplementary file 7 — Additional file 7: Fig. S5. The average temperature of every month (A) and the average precipitation of every month (B) of the studied populations of the distribution of Opisthopappus. [file 12862_2021_1790_MOESM7_ESM.docx]

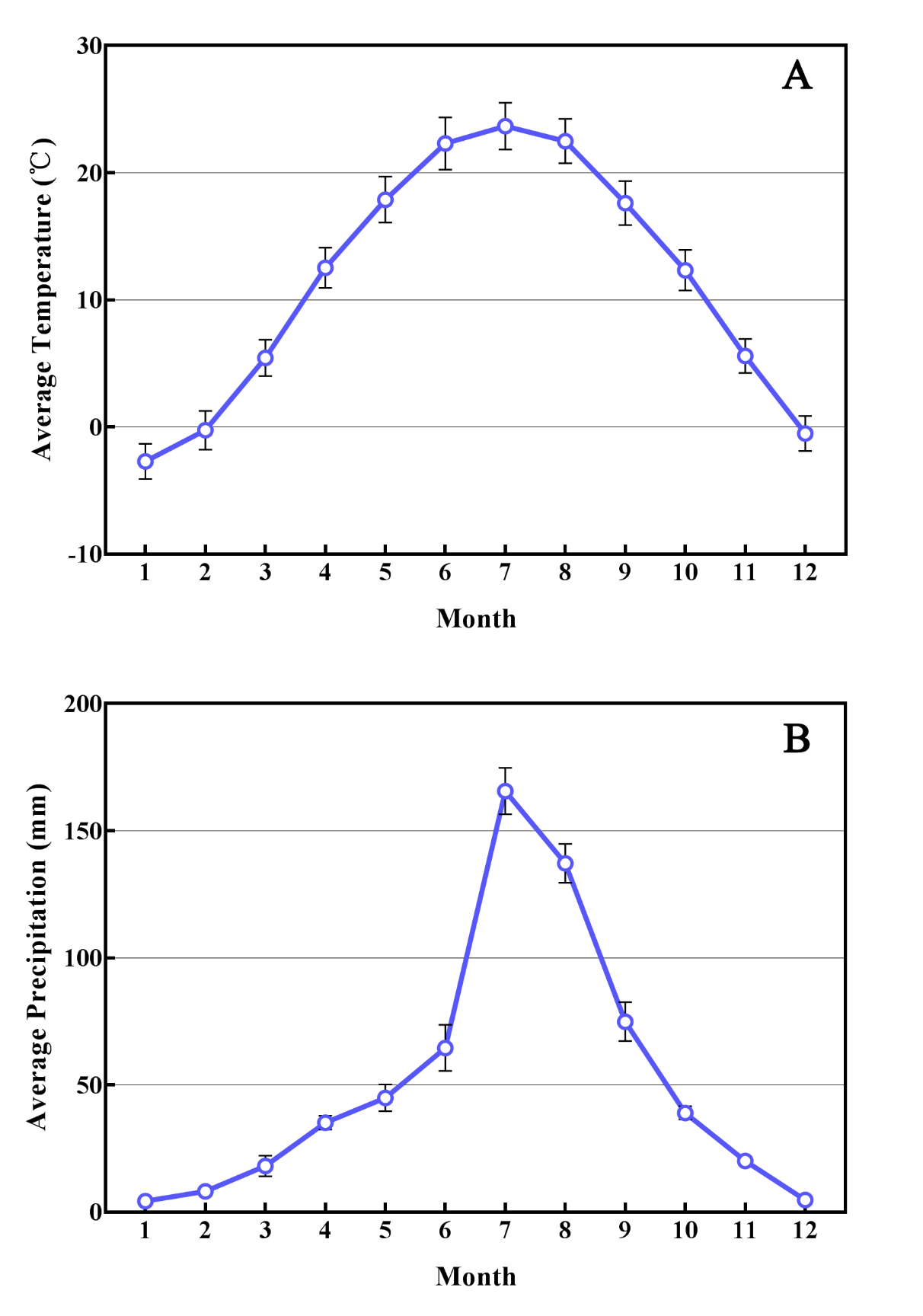


**Additional file 7: Fig. S5** The average temperature of every month **(A)** and the average precipitation of every month **(B)** of the studied populations of the distribution of *Opisthopappus.*
