# Supplementary material for: Localized environmental heterogeneity drives the population differentiation of two endangered and endemic Opisthopappus Shih species
Source: BMC Ecol Evol. 2021 Apr 15;21:56. doi: 10.1186/s12862-021-01790-0 (PMC8050911; doi:10.1186/s12862-021-01790-0)
Supplement: Supplementary file 8 — Additional file 8: Table S3. Information of primer pairs. [file 12862_2021_1790_MOESM8_ESM.docx]

| Additional file 8: Table S3. Information of primer pairs | | | |
| --- | --- | --- | --- |
| Number | Primer name | Primers sequences 5’-3’ | AT (℃) |
| 1 | SNP2 | A : CAACTTGTTAGGGTCATCCA | 53 |
|  |  | S : TGTCCGAGGTTTATTTAGGG |  |
| 2 | SNP4 | A : GGCAACCATGATCCACCAGC | 61 |
|  |  | S : GGACGGATTCATCGGTAGGG |  |
| 3 | SNP13 | A : CACTCAATCGCACTCCTTTC | 55 |
|  |  | S : CGCTCGTAGTAATCGCAGAC |  |
| 4 | SNP19 | A : TGTTCAACGTCATCGCCCTA | 60 |
|  |  | S : CCCCTGATAAAGTCGCCTCG |  |
| 5 | SNP23 | A : AACCCAACAGGATCATTCG | 55 |
|  |  | S :ATCACTCATTTCCGCAAACCTAA |  |
| 6 | SNP26 | A : CAAGTTCCTCCTCAAATCCC | 56 |
|  |  | S : TGTCCGTCACCGTTACCCTA |  |
| 7 | SNP29 | A : CATTGATGGTCGTTAGGTTTG | 53 |
|  |  | S : AATACTTGAGCCGAGTTTGC |  |
| 8 | SNP32 | A : TGGTGACTACGGGTGGGACA | 54 |
|  |  | S : CCTGAACAAAGAAACCGAACA |  |
| 9 | InDel01 | A : TCTTCTGACATGACTGCT | 55 |
|  |  | S : ACTCTTGCTTTGGTGGCT |  |
| 10 | InDel02 | A : CAGTTTCAAGACGGCATT | 52 |
|  |  | S : CCCACCACCCTAAGACAT |  |
| 11 | InDel03 | A : GATTCAAGTTCGCTGCCTAT | 55 |
|  |  | S : GAAGAATGGAGGGATGAC |  |
| 12 | InDel04 | A : GTCTCAACCACTTCACCC | 56 |
|  |  | S : TTGCCCTTGCTCGTACTCT |  |
| 13 | InDel05 | A : GAGAGGTGCATCTTGTTG | 51 |
|  |  | S : AGACGATGTCCGACTCAA |  |
| 14 | InDel06 | A : CTGCTGCTTCCACTATTGCT | 56 |
|  |  | S : CCCTCCTCCCTCTTGTTT |  |
| 15 | InDel07 | A : GTGCCTGTAGTCTCCATTAG | 54 |
|  |  | S : TTTCACCGCCATTTGTCT |  |
| 16 | InDel08 | A : ACCGCTCAATCGTACTAAAC | 53 |
|  |  | S : TGAAGAACTCAAACCCTAAC |  |
| 17 | InDel09 | A : CCGCCAGATGGTCAGTTA | 57 |
|  |  | S : CCCGTTGTTTCGGCTCTA |  |
| 18 | InDel10 | A : AACACCACATCCCTCACAGA | 56 |
|  |  | S : CCTAATAAGGCTCTACCCAA |  |

1-8 for SNP and 9-18 for InDel
